# Neuronal dysfunction caused by FUSR521G promotes ALS-associated phenotypes that are attenuated by NF-κB inhibition

**DOI:** 10.1186/s40478-023-01671-1

**Published:** 2023-11-16

**Authors:** Mari Carmen Pelaez, Antoine Desmeules, Pauline A. Gelon, Bastien Glasson, Laetitia Marcadet, Alicia Rodgers, Daniel Phaneuf, Silvia Pozzi, Paul A. Dutchak, Jean-Pierre Julien, Chantelle F. Sephton

**Affiliations:** Department of Psychiatry and Neuroscience, CERVO Brain Research Centre, Laval University, Quebec City, QC Canada

**Keywords:** Amyotrophic lateral sclerosis (ALS), Frontotemporal dementia (FTD), Motor neuron disease (MND), Synapse, Dendrite, Synaptopathy, Fused in sarcoma (FUS), Mitochondria, Cell-autonomous, Nuclear factor kappa B (NF-κB)

## Abstract

Amyotrophic lateral sclerosis (ALS) and frontotemporal dementia (FTD) are related neurodegenerative diseases that belong to a common disease spectrum based on overlapping clinical, pathological and genetic evidence. Early pathological changes to the morphology and synapses of affected neuron populations in ALS/FTD suggest a common underlying mechanism of disease that requires further investigation. Fused in sarcoma (FUS) is a DNA/RNA-binding protein with known genetic and pathological links to ALS/FTD. Expression of ALS-linked FUS mutants in mice causes cognitive and motor defects, which correlate with loss of motor neuron dendritic branching and synapses, in addition to other pathological features of ALS/FTD. The role of ALS-linked FUS mutants in causing ALS/FTD-associated disease phenotypes is well established, but there are significant gaps in our understanding of the cell-autonomous role of FUS in promoting structural changes to motor neurons, and how these changes relate to disease progression. Here we generated a neuron-specific FUS-transgenic mouse model expressing the ALS-linked human FUSR521G variant, hFUS^R521G/Syn1^, to investigate the cell-autonomous role of FUSR521G in causing loss of dendritic branching and synapses of motor neurons, and to understand how these changes relate to ALS-associated phenotypes. Longitudinal analysis of mice revealed that cognitive impairments in juvenile hFUS^R521G/Syn1^ mice coincide with reduced dendritic branching of cortical motor neurons in the absence of motor impairments or changes in the neuromorphology of spinal motor neurons. Motor impairments and dendritic attrition of spinal motor neurons developed later in aged hFUS^R521G/Syn1^ mice, along with FUS cytoplasmic mislocalisation, mitochondrial abnormalities and glial activation. Neuroinflammation promotes neuronal dysfunction and drives disease progression in ALS/FTD. The therapeutic effects of inhibiting the pro-inflammatory nuclear factor kappa B (NF-κB) pathway with an analog of Withaferin A, IMS-088, were assessed in symptomatic hFUS^R521G/Syn1^ mice and were found to improve cognitive and motor function, increase dendritic branches and synapses of motor neurons, and attenuate other ALS/FTD-associated pathological features. Treatment of primary cortical neurons expressing FUSR521G with IMS-088 promoted the restoration of dendritic mitochondrial numbers and mitochondrial activity to wild-type levels, suggesting that inhibition of NF-κB permits the restoration of mitochondrial stasis in our models. Collectively, this work demonstrates that FUSR521G has a cell-autonomous role in causing early pathological changes to dendritic and synaptic structures of motor neurons, and that these changes precede motor defects and other well-known pathological features of ALS/FTD. Finally, these findings provide further support that modulation of the NF-κB pathway in ALS/FTD is an important therapeutic approach to attenuate disease.

**Supplementary Information:**

The online version contains supplementary material available at 10.1186/s40478-023-01671-1.

## Introduction

Amyotrophic lateral sclerosis (ALS) is the most common adult-onset motor neuron disease, characterised by loss of voluntary movement resulting from degeneration of cortical and spinal motor neurons. Nearly 50% of ALS cases also meet the diagnosis criteria for frontotemporal dementia (FTD), which is marked by neuronal loss in the frontal and temporal lobes that contribute to behavioural changes and cognitive abnormalities [1–3]. Among FTD subtypes, ~ 40% of patients with behavioral variant frontotemporal dementia (bvFTD) develop motor neuron dysfunction and meet the criteria for motor neuron disease (FTD-MND) [1, 4]. Similarly, ALS and FTD share the pathological feature of aggregated proteins such as dipeptide repeats (DPRs) that are transcribed from the hexanucleotide repeat expansion within intron 1 of *chromosome 9 open reading frame 72* (*C9ORF72*) [5, 6], TAR-DNA binding protein (TDP-43) [7, 8] and fused in sarcoma (FUS) [9–11]. While the majority of ALS and FTD are considered sporadic, 10–15% of ALS and 10–20% of FTD are identified as familial with an inherited genetic risk factor [12]. To date, ALS and FTD share at least 17 gene susceptibilities including those for *C9ORF72*, *TARDBP* (or *TDP-43*) and *FUS* [12]. Over 50 mutations have been identified in the *FUS* gene and account for approximately ~ 5–10% of familial ALS and ~ 0.4% of sporadic ALS [9, 10]. Whereas, only a few *FUS* mutations have been identified in familial FTD [13, 14]. ALS and FTD-linked *FUS* mutations result in pathological aggregation of FUS, with varying levels of ubiquitination, in the cytoplasm and nucleus of neurons and glia throughout the affected regions of the cortex, brainstem and spinal cord [9, 10, 15–19]. Based on the overlap of clinical, pathological and genetic features of ALS and FTD, these diseases are considered to be part of a common disease spectrum [12]. While significant advances have been made in our understanding of ALS/FTD, there is no consensus as to how these diseases are initiated, nor are there effective therapeutic strategies for treating the vast majority of ALS/FTD cases.

Pathological changes to dendritic and synaptic structures are common features of all forms of ALS/FTD [12]. Post-mortem analysis of motor neurons from ALS and FTD cases show dendritic attrition and thinning of dendritic branches in the apical and basal dendrites of cortical motor neurons [20–23] as well as lower motor neurons of patients with motor impairments [24]. Consistent with changes in dendritic branching, alterations in synaptic densities are also observed in ALS/FTD [22, 25–30]. In ALS, the pre-synaptic densities of cortical motor neurons are reported to be unchanged, however, the number of post-synaptic densities are significantly reduced [22, 25]. These findings are consistent with a significant loss of pre-synaptic densities around the soma and proximal dendrites of lower motor neurons in ALS spinal cord tissues [26]. In line with these studies, proteomic and transcriptomic research of human ALS and FTD tissues show that changes in synaptic proteins and genes are affected in disease [31–34]. Additionally, synaptic loss is found to correlate with disease severity in ALS and FTD. Analysis of prefrontal cortex from sporadic ALS patients showed that the severity of cognitive impairments correlated with the amount of synaptic loss and were not due to cortical atrophy or dementia-associated neuropathology [25]. Similarly, in vivo analysis of FTD patients with *C9ORF72* hexanucleotide repeat expansions by positron emission tomography (PET) scan revealed reduced synaptic densities in the thalamus of pre-symptomatic and extensive synaptic loss in the frontotemporal regions of symptomatic patient [35]. Importantly, these findings indicate that cortical synaptic loss occurs early in the pre-symptomatic stages of the disease.

Animal models of ALS/FTD exhibit reduced dendritic branching and synaptic loss of motor neurons [36–41], including models harbouring FUS mutants [42]. FUS is a multi-functional DNA/RNA binding protein involved in transcription and several aspects of RNA metabolism [43–54]. The majority of *FUS* mutations identified in familial ALS are found mainly within the C-terminal PY-NLS region of the encoded protein [9, 10] and cause mislocalisation of this predominantly nuclear protein to the cytoplasm [55, 56]. The consequences of these changes are shown to affect transcription, mRNA metabolism and proteostasis, which in turn affect several aspects of cellular status and function [57–60]. Indeed, global transgenic mice expressing low levels of human FUSR521G develop motor and cognitive deficits that correlate with reduced dendritic branching of the sensorimotor cortex and lower motor neurons, loss of synapses and activation of astrocytes and microglia in the brain and spinal cord [42]. Neuron-specific expression of ALS-linked FUS mutants in models of disease show similar changes in neuromorphology and synaptic loss [61–64] that impact neuronal function [63–65]. These studies highlight the importance of FUS in regulating neuronal status and the potential impact of ALS-linked FUS mutants in the central nervous system. However, there are still significant gaps in our understanding of the cell-autonomous and non-cell autonomous role of FUS mutants in promoting structural changes to motor neurons, and how these changes relate to disease progression.

In the present study, we investigated the cell-autonomous effects of the ALS-linked FUS R521G variant on dendritic branches and synapses of motor neurons and examined how these changes relate to ALS-associated pathology. We generated a neuron-specific mouse model expressing human FUSR521G, hereafter referred to as hFUS^R521G/Syn1^ and found that cognitive defects and reduced dendritic branching of cortical motor neurons occurred in 1-month-old mice in the absence of synaptic loss and other pathological markers of ALS/FTD. Longitudinal analysis of hFUS^R521G/Syn1^ mice showed that cognitive defects persisted and by 6 months of age displayed motor impairments that corresponded with dendritic attrition of lower motor neurons and synaptic loss. In addition to these changes, 6-months-old hFUS^R521G/Syn1^ mice displayed cytoplasmic mislocalisation of FUSR521G, reduced mitochondrial expression of translocase of outer mitochondrial membrane 20 (TOM20) indicative of mitochondrial dysfunction [66], and activation of astrocytes and microglia. Neuroinflammation and glial activation are shown to promote neuronal dysfunction and drive disease progression in ALS/FTD [67, 68]. The therapeutic effects of IMS-088, an inhibitor of the canonical NF-κB pathway [69–71], were assessed in hFUS^R521G/Syn1^ mice and found to improve cognitive and motor function and promote the restoration of dendritic branches and synapses of motor neurons. Moreover, treatment of hFUS^R521G/Syn1^ mice with IMS-088 attenuated glial activation and restored nuclear FUSR521G localisation and mitochondrial expression of TOM20 in neurons. In vitro studies showed that treatment of primary cortical neurons expressing FUSR521G with IMS-088 promoted an increase in dendritic mitochondrial numbers and mitochondrial activity to similar levels found in wild-type cultures, suggesting that inhibition of NF-κB has a therapeutic effect on mitochondrial stasis in our FUSR521G models. Collectively, this work demonstrates that FUSR521G has a cell-autonomous role in causing early pathological changes to dendritic and synaptic structures of motor neurons, and that these changes precede motor defects and other well-known pathological features of ALS/FTD. Additionally, we found that neuron-restricted expression of FUSR521G promotes non-cell autonomous effects on glial activation in hFUS^R521G/Syn1^ mice. Finally, our findings suggest that targeting the canonical NF-κB pathway is a potential therapeutic strategy for stabilizing dendritic structures and maintaining corticospinal connectivity in patients with ALS/FTD.

## Materials and methods

### Mouse models

All animal studies were carried out using protocols approved by the Canadian Council on Animal Care (CCAC) and by the Université Laval Committee on Ethics and Animal Research. Human FUSR521G/Syn1Cre (hFUS^R521G/Syn1^) mice were generated by crossing *CAG-Z-FUSR521G-IRES-EGFP* (strain #682) mice [42] with *Tg(Syn1-cre)671Jxm* (strain # 003966), where the transgenic expression of Cre-recombinase in these mice is regulated under the control of the Synapsin 1 promoter [72], to generate neuron-specific hFUS^R521G/Syn1^ transgenic mice. hFUS^R521G/Meox^ mice were generated as previously described [42]. Genotypes of all animals were determined by PCR, using the following primers: *FUS:* (forward: 5′ GAC CAG GTG GCT CTC ACA TG 3′); (reverse: 5′ GTC GCT ACA GAC GTT GTT TGT C 3′) and Cre: (forward: 5′ GGA CAT GTT CAG GGA TCG CCA GGC 3′); (reverse: 5′ GCA TAA CCA GTG AAA CAG CAT TGC 3′).

### Behavioral testing

Behavioral tests were performed as previously described [42], and briefly detailed below. Longitudinal testing of mice was performed in order to capture the progressive cognitive and motor decline that is observed in ALS/FTD. In this study, the same mice were used for all time points. The number of mice tested for each experimental condition test included 14 (6M:8F) littermate controls (+ / + ; + / + , + / + ; + /Cre, Tg/ + ; + / +) and 12 (5M:7F) hFUS^R521G/Syn1^ transgenic (Tg/ + ; + /Cre) mice.

#### Novel object recognition test

This test is used to assess recognition memory [73]. On the first day of testing, mice were habituated to an empty testing arena for 5 min. On the second day, mice were presented with two identical “familiar” training objects for 5 min. After 24 h of completing the second day of training, the probe tests were carried out. The animals were exposed to the same arena, where they were presented with one familiar object and one novel object. The time of the interactions with each object were recorded for 5 min. Interactions were defined as nosing, touching, and sniffing.

#### Passive avoidance

This is a fear-aggravated test used to assess learning and memory in a fear-aggravated environment [74]. The testing apparatus consisted of light and dark compartments of equal size that are separated by a guillotine door. The first day of the test, mice were habituated in the testing arena. Each mouse was placed in the light compartment and after 30 s the door was opened, allowing the animal to cross to the dark chamber. On the second day, mice were again placed in the light compartment and after 30 s the door was opened. Immediately after entry of each mouse into the dark chamber an electrical foot shock (0.5 mA for 2 s) was delivered. On the third day of the test, each mouse was again placed in the light compartment and after 5 s the door was opened. The latency to enter the dark compartment was measured for a maximum of 5 min.

#### Hindlimb splay

This test is used to assess hindlimb strength [42]. Mice were held vertically at the mid-point of the tail 50 cm above a layer of soft bedding for a maximum of 30 s. Mice were scored as follows: 0 = both hindlimbs completely retracted; 1 = both hindlimbs partially retracted; 2 = one hindlimb retracted; 3 = hindlimbs splayed away from the abdomen.

#### Wire hanging test

This test is used to measure motor function by assessing limb strength [75]. Mice were placed on a wire 50 cm above a layer of soft bedding and the latency to fall was measured. Three attempts per mouse were made over three consecutive days for a maximum of 5 min per attempt.

#### Grip test

This test is used to measure motor function by assessing limb strength [42]. Mice were placed on a metal grid maintained horizontally 35 cm above a layer of soft bedding. The metal grid was inverted and the latency of the mice to fall was measured. The test was conducted over three consecutive days for a maximum of 3 min per attempt and three attempts per mouse.

#### Rotarod

This test is used to measure motor function [42]. Mice were placed on a stationary rotarod which was accelerated at 0.1rpm/s for a total of 5 min. The latency time to fall from the rod was recorded for each mouse. Each mouse was tested four times a day over two consecutive days with an intertrial interval of 10 min.

### IMS-088 treatment of hFUS^R521G^^/Syn1^ mice

IMS-088 was provided by IMSTAR therapeutic (Vancouver, Canada). hFUS^R521G/Syn1^ mice were treated with 30mg/kg IMS-088 or vehicle (0.9% saline with 2.5% Tween-80 and 5% DMSO) by gavage daily for 8 weeks. Behavior testing was performed during the last 12 days of the treatment period. The number of mice tested for each experimental condition test included 12 (6M:6F) vehicle treated littermate controls (+ / + ; + / + , + / + ; + /Cre, Tg/ + ; + / +), 9 (5M:4F) vehicle treated hFUS^R521G/Syn1^ transgenic (Tg/ + ; + /Cre) and 9 (4M:5F) IMS treated hFUS^R521G/Syn1^ transgenic (Tg/ + ; + /Cre) mice.

### Western blotting

Tissues were homogenized in lysis buffer containing 50 mM Tris pH8.0, 140 mM NaCl, 1 mM EDTA pH8, 1% Triton X-100, 0.1% Na-deoxycholate supplemented with fresh 1 mM DTT, protease inhibitors (Sigma, 11,836,170,001) and 1X PHosSTOP (Sigma, 04906845001). Lysates were incubated for 30 min on ice and centrifugated at 18 000×*g* for 25 min at 4 °C. Cleared lysates were collected and boiled in 1X Laemmli buffer, 5 min at 95 °C. Protein samples were separated on a 10% SDS-PAGE and transferred to nitrocellulose membranes for Western blotting as previously described [18, 54]. Membranes were blocked with 5% non-fat dried skim milk in Tris-buffered saline containing 0.1% (w/v) Tween 20 (TBST) for 1h at room temperature (RT), and incubated with primary antibodies (Table 1) overnight at 4°C. After washing three times for 10 min with TBST, the membranes were incubated with fluorescent LI-COR secondary antibodies for 1 h at RT, washed three times for 10 min with TBST and imaged using the LI-COR Odyssey imaging system.

**Table 1:** List of antibodies

Antibody	Species	Dilution	Company	Catalog no
Acetylated p65 (acetyl K310)	Rabbit	1:1000	Abcam	ab19870
Alpha-bungarotoxin-647 conjugate	Mouse-Conjugated	1:500	Thermo scientific	B35450
Beta-Actin	Mouse	1:30,000	Cell Signaling Technology	3700
CD11b	Rat	1:200	BioRad	MCA711
ChAT	Goat	1:500	Sigma-Aldrich	AB144p
GFAP	Chicken	1:500	Sigma-Aldrich	AB5541
Human FUS	Rabbit	1:500	Produced by Yu lab [42]	N/A
Iba1	Rabbit	1:500	Wako	019-19741
MAP2	Chicken	1:500	Abcam	AB5392
MAP2	Mouse	1:500	Sigma-Aldrich	MAB3418
NeuN	Mouse	1:500	Sigma-Aldrich	MAB377
Neurofilament light	Mouse	1:1000	Cell Signaling Technology	2835
SV2	Mouse	1:1000	DSHB	SV2
Synaptophysin	Mouse	1:500	Synaptic System GmbH	101–011
TOM20	Rabbit	1:500	Santa Cruz Biotechnology	sc-11415
Total FUS	Rabbit	1:500	Sigma-Aldrich	HPA008784
Alexa Fluor 488 goat anti-chicken	Chicken	1:500	Abcam	ab150169
Alexa Fluor 488 goat anti-mouse	Mouse	1:500	Life technologies	A11001
Alexa Fluor 488 goat anti-rabbit	Rabbit	1:500	Life technologies	A11034
Alexa Fluor 546 goat anti-mouse	Mouse	1:500	Life technologies	A11030
Alexa Fluor 546 goat anti-rabbit	Rabbit	1:500	Life technologies	A11035
Alexa Fluor 647 goat anti-mouse	Mouse	1:500	Life technologies	A21236
Alexa Fluor 647 goat anti-rabbit	Rabbit	1:500	Life technologies	A21245

### Primary neuron cultures

Dissociated cortical neurons were prepared from neonatal hFUS^R521G/Meox^ and littermate control pup (P0–P2) cortices as described previously [18]. Cells were plated at a density of 50 cells/mm^2^ on glass coverslips laminin-coated on a PDL layer (Neuvitro Corporation). Complete Neurobasal media was supplemented with serum-free B-27™ (50:1; Gibco, 17,504,001), penicillin/streptomycin (50 U/mL; 50 μg/mL; Gibco, 15,140,148) and 0.5 mM L-GlutaMAX (Invitrogen, 35,050,061). When plating cells, fetal bovine serum (5%; Hyclone SH30071.03) was added to complete Neurobasal media. To limit the proliferation of non-neuronal cells, cultures 5 days in vitro (DIV5) received a half media change of serum-free growth medium containing Ara-C (5 µM; Sigma, C1768). Neurons were fed twice a week by replacing half of the conditioned medium with fresh complete Neurobasal media. Treatment of primary cultured neurons with Vehicle (1X PBS) or IMS-088 (1 µM, 2h) occurred at 14–15 days in vitro (DIV). Mitotracker Orange (100 nM, CMTMRos, M7510) was added to the culture media 30 min before the end of the treatments and incubated at 37 °C.

### Immunofluorescence

Mice were anesthetized with ketamine/xylazine (100mg/kg/10mg/kg) and perfused with 0.9% saline and 4% paraformaldehyde (PFA). Brain and spinal cord were post-fixed in 4% PFA for 24 h at 4°C, then transferred to a 30% (w/w) sucrose solution for 24–48 h at 4 °C. Tissues were sectioned to a 40 µm thickness using a microtome (Microm HM430, ThermoFisher). Tissue sections were washed three times with 1X PBS for 10 min, followed by a 1 h incubation in blocking solution containing 3% BSA, 5% normal goat serum (NGS), 0.3% TritonX-100, 0.02% NaAz in 1X PBS. Sections were incubated overnight at 4 °C in primary antibodies (Table 1) diluted in incubation solution containing 1% BSA, 3% NGS, 0.3% Triton X-100, 0.02% NaAz in 1X PBS. The next day, sections were washed three times with 1X PBS, followed by a 2 h incubation at RT with Alexa-Fluor-conjugated secondary antibodies (Table 1) diluted in incubation solution. Sections were washed three times for 10 min with 1X PBS and mounted in Vectashield mounting media (Vector Laboratories, VECTH1200). Imaging of the motor cortex and ventral horn of the spinal cord was performed using a Zeiss LSM710 inverted confocal and imaged as z-stacks (1–2 µm steps per stack) from 3–4 biological replicates per group. Maximum intensity projection images for 5–7 images per biological replicate were analysed in Fiji ImageJ using the signal intensity measuring tool. No background subtraction was applied in the analysis and all normalization was performed relative to littermate control mice.

Motor neuron analysis was performed on lumbar spinal cord sections stained with anti-choline acetyltransferase (ChAT) and anti-NeuN (Table 1) to label cholinergic motor neurons. Confocal images were captured as described above and motor neurons were classified as being ChAT and NeuN positive and with a cell body diameter > 100 µm. Cell body diameter of motor neurons was determined using the Fiji ImageJ selection tool. 7–10 tissue sections were quantified per animal from 3–4 biological replicates per group.

Neuromuscular junction (NMJ) staining was performed on gastrocnemius muscle isolated from PFA-perfused mice. Muscles were frozen, embedded with O.C.T and longitudinally cut at a 30 µm thickness using a cryostat (Thermo Scientific NX50). Sections were incubated with primary antibodies synaptophysin and neurofilament light (Table 1) overnight at 4 °C. The next day, sections were washed three times with 1X PBS for 5 min each, followed by an incubation with secondary antibodies and alpha-bungarotoxin (Table 1) for 2 h at RT. Sections were then washed three times with 1X PBS and mounted with Vectashield mounting media. NMJs images were obtained by confocal microscopy described above. NMJs were manually classified as innervated, partially denervated and totally denervated as previously described [42], from 20 to 50 individual NMJs from 3 to 5 biological replicates per group.

Primary cortical neurons (DIV14-15) were fixed with 4% PFA solution pre-warmed at 37 °C for 10 min at RT. Fixed cells were washed three times with 1X PBS for 5 min each and then incubated for 30 min with a blocking solution containing 10% NGS, 0.2% Triton X-100, 0.1% NaAz in 1X PBS. Cells were then incubated with primary antibodies (Table 1) diluted in blocking/permeabilization solution at 4 °C overnight. Cells were washed three times with 1X PBS, followed by 1 h incubation at RT with secondary antibodies diluted in blocking/permeabilization solution. Cells were washed three times with 1X PBS and then mounted with ProLong Gold Antifade mounting media containing DAPI (Thermo Fisher, P3635). Z-stack confocal images of neurons were obtained by confocal microscopy as described above. Mitochondria were labelled using Mitotracker Orange or TOM20 and analysed in IMARIS using the spot-detection function. An arbitrary mean quality threshold was used to detect mitochondria. The number, volume and area of the different mitochondria were calculated and presented as mean per cell analysed (for Mitotracker Orange) or mean per soma analysed (for TOM20). Analysis of mitochondria in the cell body and dendrites was performed by manual selection using MAP2 from 18 individual neurons from 3 to 4 biological replicates per group.

### TMRM live-cell imaging on mice cortical neurons

Primary cortical neurons were plated at 50 cells/mm^2^ on glass coverslips (Ibidi USA, #80,426) that were laminin-coated on a PDL layer. At DIV14-15 neurons were treated with vehicle or IMS-088. Tetramethylrhodamine, methyl ester (TMRM, 50 nM, ThermoFisher, # I34361), a red–orange fluorescent dye that is readily sequestered by active mitochondria, was added to the cultures 30 min before live-cell imaging. Cultures were washed in 1X PBS and media was replaced with conditioned neurobasal media before z-stack images (1 µm step per stack) were captured at a constant temperature of 37 °C and 5.0% of CO_2_ using a Zeiss LSM710 inverted confocal. TMRM signal intensity was analysed as described above from 8 to 15 individual neurons from 3–4 biological replicates per group.

### Golgi staining and morphological analysis

Mouse brain and spinal cords were used for Golgi staining using a kit (FD Rapid GolgiStain™ kit) according to the manufacturer’s instructions. Tissues were incubated at RT in A and B solutions (1:1) for 2 weeks in the dark, followed by incubation in solution C at 4 °C for 48 h. Tissues were then frozen in O.C.T, sectioned at 100 µm thickness using a Thermo Scientific Cryostat NX50, and mounted on gelatine-coated slides (1% gelatin and 0.1% chromium potassium sulfate dodecahydrate). After completing the Golgi staining according to the kit instructions, sections were covered with Permount mounting media (Fisher Scientific, SP15-100) and stored at RT in the dark until analysis.

Cortical and spinal motor neuron images were taken with an AxioImager.M2 microscope. Cortical neurons from layers IV–V of the M1 and M2 regions and motor neurons with a cell body diameter > 100 µm located at the ventral horn of the spinal cord were selected for the analysis. Motor neurons were 3D reconstructed using Neurolucida 360 software (MBF Bioscience, Williston, VT) using the ‘soma detector’ tool and the dendritic reconstruction ‘smart-manual’ option. Reconstructed neuronal tracing files were analysed in Neurolucida Explorer for Sholl analysis. Sholl analysis was performed starting a 10 µm from the cell body with 5 µm increasing intervals throughout the whole neuronal tracing.

Spine detection was performed in Neurolucida 360 in the first 100 µm of the apical dendrite of cortical motor neurons, then spines were classified according to their shape in thin, filopodia, stubby and mushroom/mature. Spine density was calculated from 30 to 100 µm distance from the soma. Sholl and spine analysis was performed on 10 individual motor neurons from 3 biological replicates per group for a total of 30 neurons.

### Statistics

All statistical analyses were performed using GraphPad Prism 8 (GraphPad, San Diego, CA). One-way and two-way ANOVA analyses used the Bonferroni post-hoc test. All values given in the text and figures indicate mean ± standard error of the mean (SEM). The level of significance was specified as follows: * *p* < 0.05, ** *p* < 0.01, ***p < 0.005 and **** *p* < 0.001.

## Results

### Neuronal expression of FUSR521G causes cell-autonomous defects in neuromorphology that precede motor impairment

**Fig. 1:**
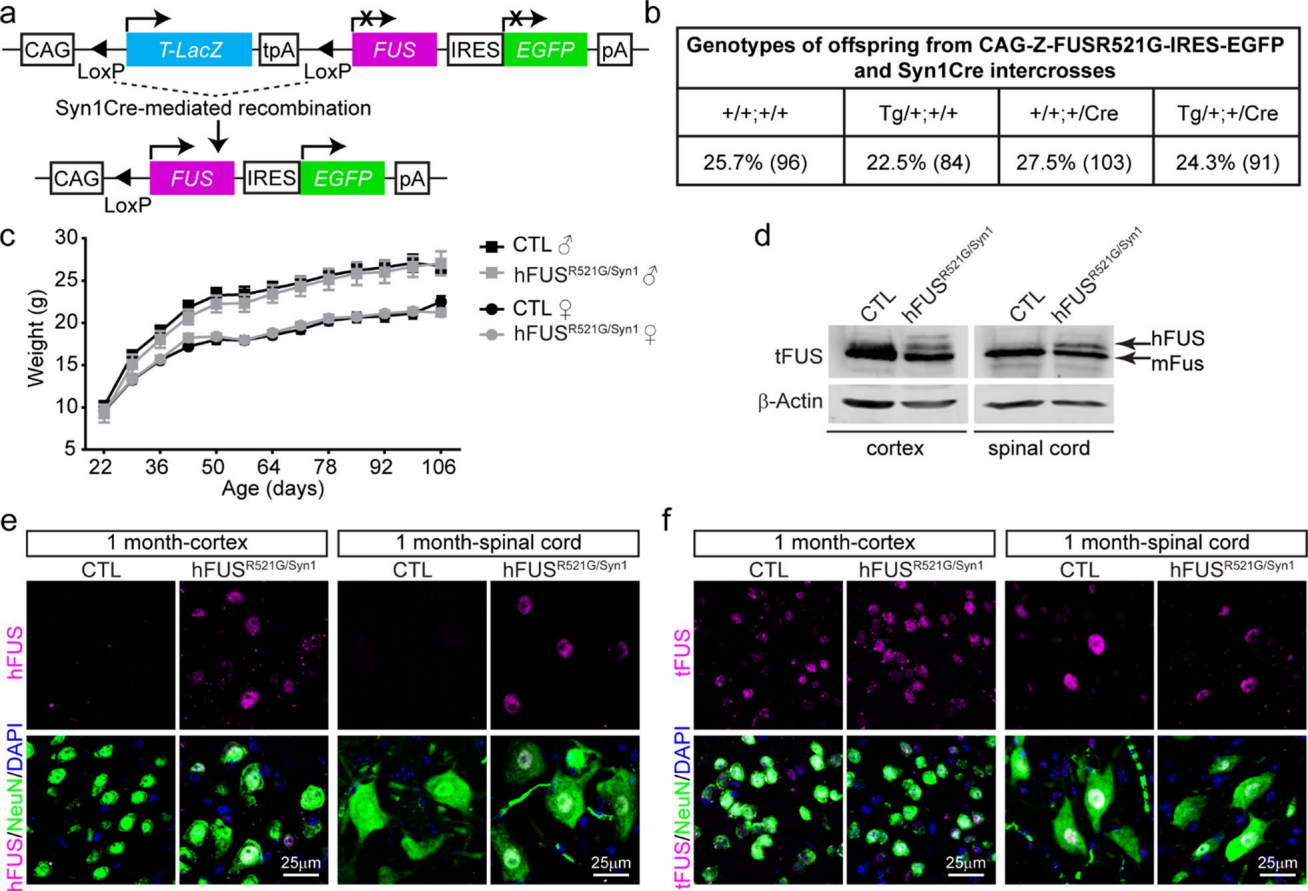
Generation of neuron-specific hFUS^R521G/Syn1^ mice. **a** Schematic of human FUSR521G/Syn1Cre (hFUS^R521G/Syn1^) mice generation. **b** Mendelian ratios of offspring genotypes from 56 intercrosses between CAG-Z-FUSR521G-IRES-EGFP and Syn1Cre mice. The numbers indicated in brackets are the number of mice for each genotype. **c** Weight curve of hFUS^R521G/Syn1^ and littermate control mice 6–8 mice per group. **d** Immunoblot of lysates from indicated tissues of 1-month-old littermate control (CTL) and hFUS^R521G/Syn1^ mice. Proteins are detected with antibodies against total FUS (tFUS) and the loading control β-Actin. The lower molecular weight band in the immunoblot corresponds with endogenous mouse FUS (mFus). The higher molecular weight band in the immunoblot corresponds with human FUSR521G protein (hFUS) expressed in the brain and spinal cord tissues of hFUS^R521G/Syn1^ mice. The brain and spinal cord of CTL and hFUS^R521G/Syn1^ mice stained with anti-NeuN (neuron marker), DAPI (nuclear marker) and **e** anti-hFUS or **f** anti-tFUS. hFUS is only detected in NeuN positive cells in the cortex and spinal cord of FUS transgenic mice

Autosomal dominant *FUS* mutations are associated with familial forms of ALS/FTD [9, 10]. Previously, we established and characterised the CAG-Z-FUSR521G-IRES-EGFP transgenic mouse model, in which the human *FUSR521G* transgene is conditionally expressed when crossed with a mouse line containing Cre recombinase [42]. This work showed that global expression of human FUSR521G at low levels in mice caused cognitive and motor defects, dendritic attrition, synaptic loss and glial activation resembling ALS/FTD [42]. To investigate the cell-autonomous effects of expressing FUSR521G in neurons and this contribution to ALS/FTD-associated phenotypes, the CAG-Z-FUSR521G-IRES-EGFP transgenic mice were crossed with Syn1Cre mice (Fig. 1a), hereafter referred to as hFUS^R521G/Syn1^. hFUS^R521G/Syn1^ mice were born at normal Mendelian ratios (Fig. 1b) and showed no significant differences in weight compared with littermate controls (Fig. 1c). FUSR521G expression was found to be restricted within the nucleus of NeuN-positive neurons and distributed evenly throughout the brain and spinal cord of hFUS^R521G/Syn1^ mice (Fig. 1d–f and Additional file 1: Figs. S1 and S2). These findings confirm the neuronal restricted expression of FUSR521G in this model.

**Fig. 2:**
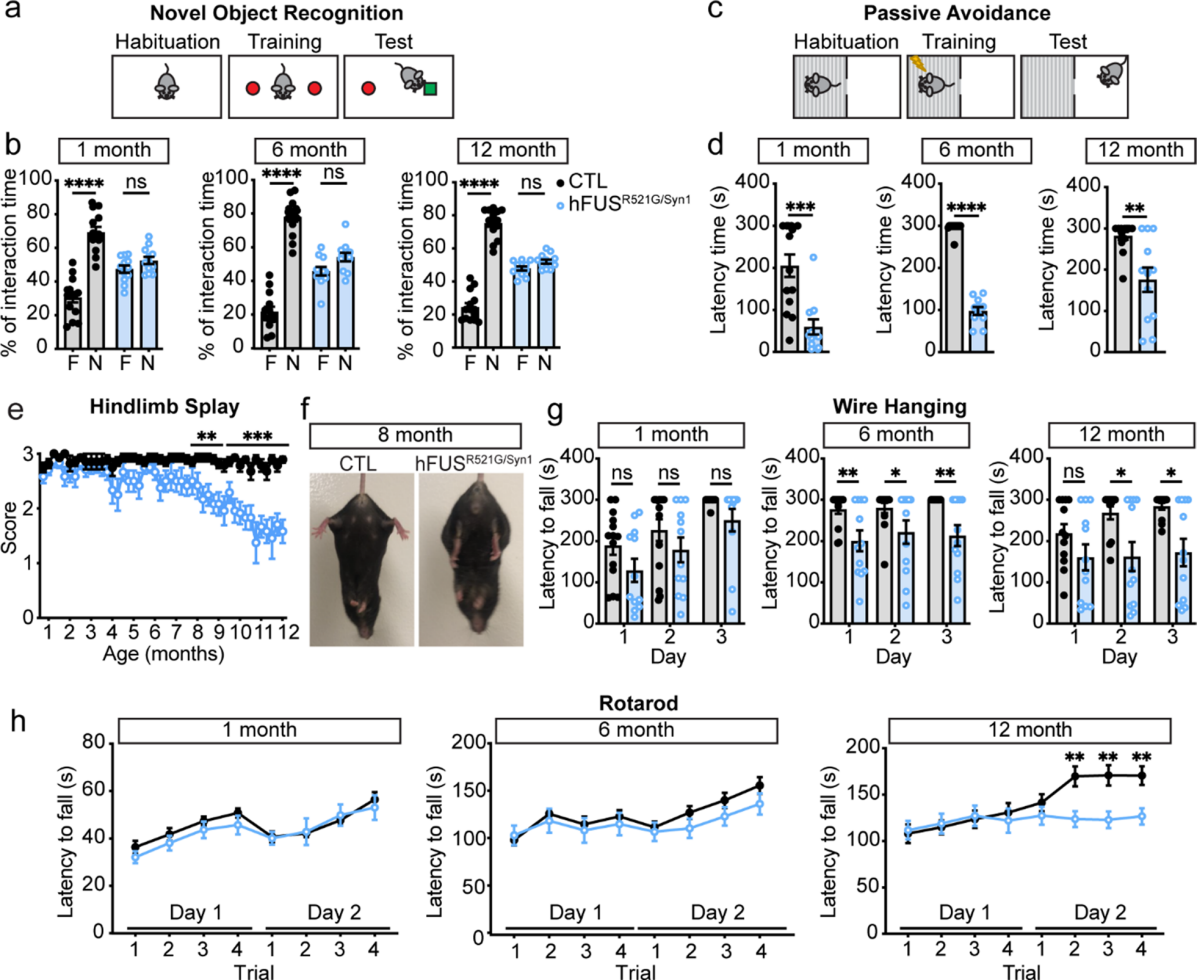
Neuronal expression of FUSR521G causes cell-autonomous defects in cognitive and motor function. **a** Schematic of the novel object recognition test. **b** hFUS^R521G/Syn1^ mice display significantly lower interaction time with the novel object (N) compared to the familiar object (F), and littermate control (CTL) mice spend more interaction time with the novel object. **c** Schematic of the passive avoidance test. **d** hFUS^R521G/Syn1^ mice display reduced latency time in entering the dark compartment compared to CTL mice. **e** hFUS^R521G/Syn1^ mice display motor impairments in hindlimb splay at 8 months of age compared to CTL mice. **f** Image shows impaired hindlimb splay of hFUS^R521G/Syn1^ mice. **g** hFUS^R521G/Syn1^ mice display grip weakness in the wire hanging test at 6 and 12 months of age compared to CTL mice. **h** hFUS^R521G/Syn1^ mice display signs of motor impairments in the rotarod test at 6 months and significant motor impairments at 12 months of age compared to CTL mice. Values from each group are expressed as mean ± SEM. Statistics uses an unpaired Student’s *t*-test for comparison between two groups (n = 12 hFUS^R521G/Syn1^ and n = 14 CTL mice/group). *p < 0.05, **p < 0.01, ***p < 0.005 ****p < 0.001, and not significant (ns)

Loss of motor function and cognitive deficits are reported in ~ 50% of ALS cases [1–3]. Behavior analysis of hFUS^R521G/Syn1^ mice was conducted longitudinally to assess the effects of neuronal restricted expression of FUSR521G on motor and cognitive function. Beginning at 1 month of age, hFUS^R521G/Syn1^ mice were assessed for cognitive function using novel object recognition and passive avoidance tests. hFUS^R521G/Syn1^ mice had significant cognitive impairments compared to littermate controls (Fig. 2a–d). Motor function was also assessed using the hindlimb splay, wire hanging and rotarod tests, but no motor impairments were observed in 1-month-old mice (Fig. 2e–h). Cognitive impairments persisted throughout the longitudinal analysis of the hFUS^R521G/Syn1^ mice (Fig. 2a–d and Additional file 1: Fig. S3a and S3b). By 6 months of age, hFUS^R521G/Syn1^ mice displayed modest motor deficits (Fig. 2e–h) that progressed to significant motor impairment by 8 months of age and worsened with age (Fig. 2e–h and Additional file 1: Fig. S3c–f). No sex-differences in behavior were observed in hFUS^R521G/Syn1^ mice (Additional file 1: Fig. S4). Severe motor impairments observed in 12-months-old mice also corresponded with a significant loss of motor neurons in the ventral spinal cord along with denervation of neuromuscular junctions (NMJs) (Additional file 1: Fig. S5). These findings indicate that neuron-specific expression of FUSR521G affects neuron populations involved in both cognitive and motor functions in hFUS^R521G/Syn1^ mice.

**Fig. 3:**
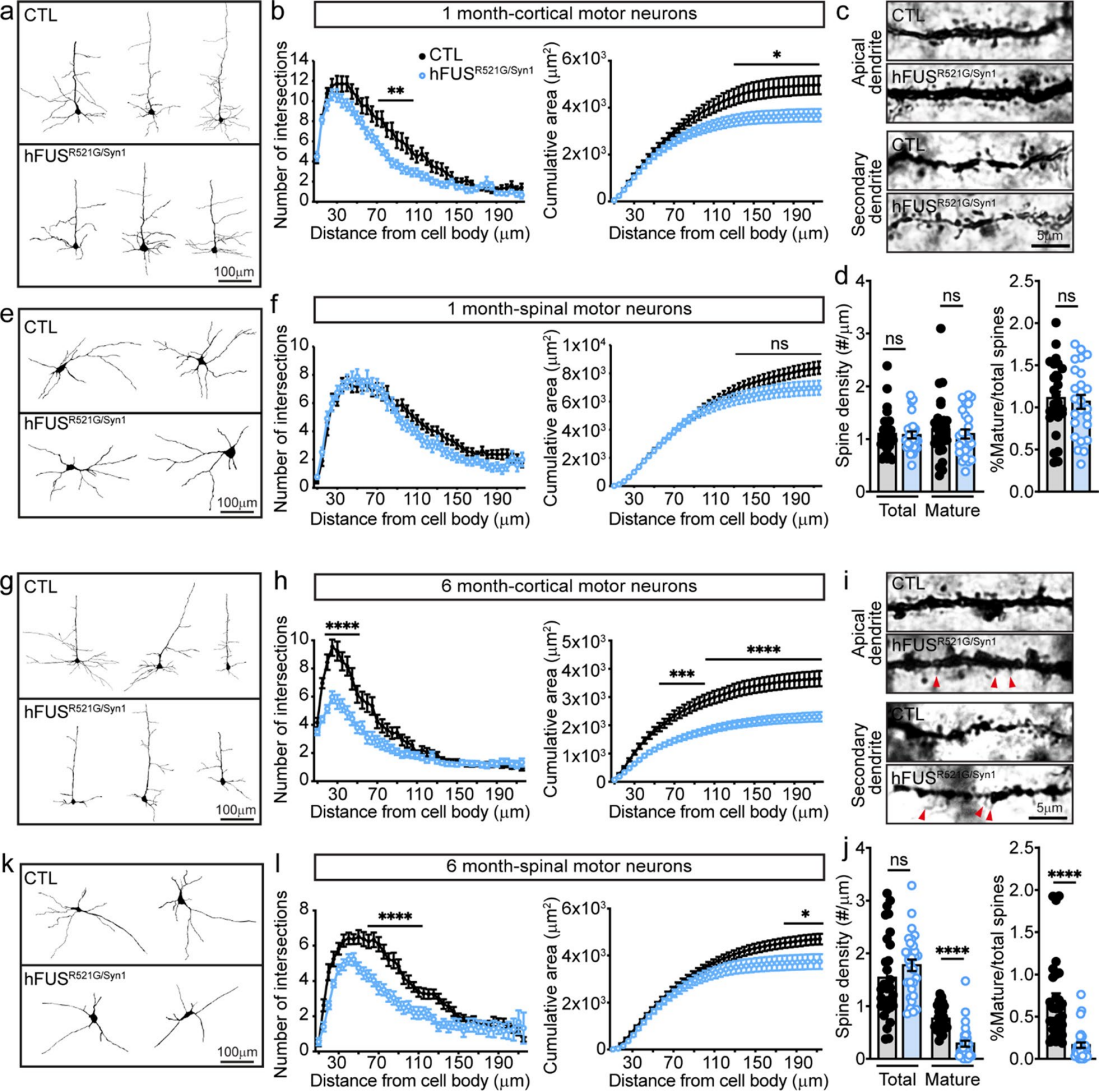
FUSR521G causes cell-autonomous defects in neuromorphology. **a** Neurolucida tracing of cortical motor neuron dendrites from 1-month-old mice. **b** Sholl analysis shows significant reductions in the dendritic intersections and cumulative area of dendrites in cortical motor neurons of 1-month-old hFUS^R521G/Syn1^ mice compared to littermate controls (CTL). **c** Golgi images of dendritic spines from layer IV-V neurons in the motor cortex from 1-month-old mice and **d** their quantification. The dendritic spine density of total and mature spines shows no differences between 1-month-old hFUS^R521G/Syn1^ and CTL mice. **e** Neurolucida tracing of spinal motor neuron dendrites from 1-month-old mice. **f** Sholl analysis shows no reduction in the dendritic intersections and cumulative area of dendrites in spinal motor neurons of 1-month-old hFUS^R521G/Syn1^ mice. **g** Neurolucida tracing of cortical motor neuron dendrites from 6-months-old mice. **h** Sholl analysis shows persistent and significant reductions in the dendritic intersections and cumulative area of dendrites in cortical motor neurons of 6-months-old FUS transgenic mice. **i** Golgi images of dendritic spines from the cortical motor cortex of 6-months-old mice and **j** their quantification. The mature dendritic spine density of 6-months-old hFUS^R521G/Syn1^ mice shows significant reduction in the number of mature spines. **k** Neurolucida tracing of the dendrites of spinal motor neurons from 6-months-old mice. **l** Sholl analysis shows significant reductions in the dendritic intersections and cumulative area of dendrites in spinal motor neurons of 6-months-old FUS transgenic mice. Values from each group are expressed as mean ± SEM. Statistics uses two-way repeat measures ANOVA for Sholl analysis and an unpaired Student’s *t*-test for comparison between two groups (n = 3–4 mice/group). *p < 0.05, **p < 0.01, ***p < 0.005 ****p < 0.001, and not significant (ns)

ALS-FUS variants cause loss of dendritic branching of motor neurons and decreased synaptic densities in ALS/FTD models of disease [42, 61–64], but it is unclear when these changes occur relative to motor decline. To address this question, the dendritic structures of cortical and spinal motor neurons of hFUS^R521G/Syn1^ mice were analysed at different stages of motor decline. In 1-month-old hFUS^R521G/Syn1^ mice, there was a significant reduction in dendritic branching and cumulative area of cortical motor neuron dendrites (Fig. 3a, b), but no changes in cortical motor neuron spine density (Fig. 3c, d). Consistent with no observed deficits in motor function in 1-month-old hFUS^R521G/Syn1^ mice (Fig. 2e–h), dendritic branches of spinal motor neurons were unaltered (Fig. 3e, f). In 6-months-old hFUS^R521G/Syn1^ mice, where motor decline is first observed (Fig. 2e–h), more significant reductions in dendritic branching (Fig. 3g, h) and fewer mature spines and a decrease in spine density are observed in cortical motor neurons (Fig. 3i, j). Significant dendritic attrition and reduction in cumulative area were observed in spinal motor neurons of these mice (Fig. 3k, l), which is consistent with motor decline at this age. However, loss of spinal motor neurons or denervation of NMJs were not detected in 6-months-old hFUS^R521G/Syn1^ mice (Additional file 1: Fig. S6).

Collectively, the analysis of dendritic branching and synapses from 1- and 6-month-old mice show that a decrease in dendritic branching of cortical motor neurons precedes synaptopathy and dendritic attrition of spinal motor neurons in hFUS^R521G/Syn1^ mice. Moreover, significant changes in dendritic branches of cortical and spinal motor neurons and loss of cortical dendritic spines coincide with the emergence of motor impairments in hFUS^R521G/Syn1^ mice. Our findings indicate that neuronal defects begin in the motor cortex of hFUS^R521G/Syn1^ mice and descend to the spinal motor neurons to promote motor impairments, which supports the hypothesis that ALS starts in the cortical motor neurons, descends to the lower motor neurons [76, 77], as opposed to the dying-back model, which proposes ALS pathology advances to the brain a retrograde direction from spinal motor neuron synaptic terminals [78].

### Neuron-specific expression of FUSR521G promotes neuropathological features of ALS/FTD

**Fig. 4:**
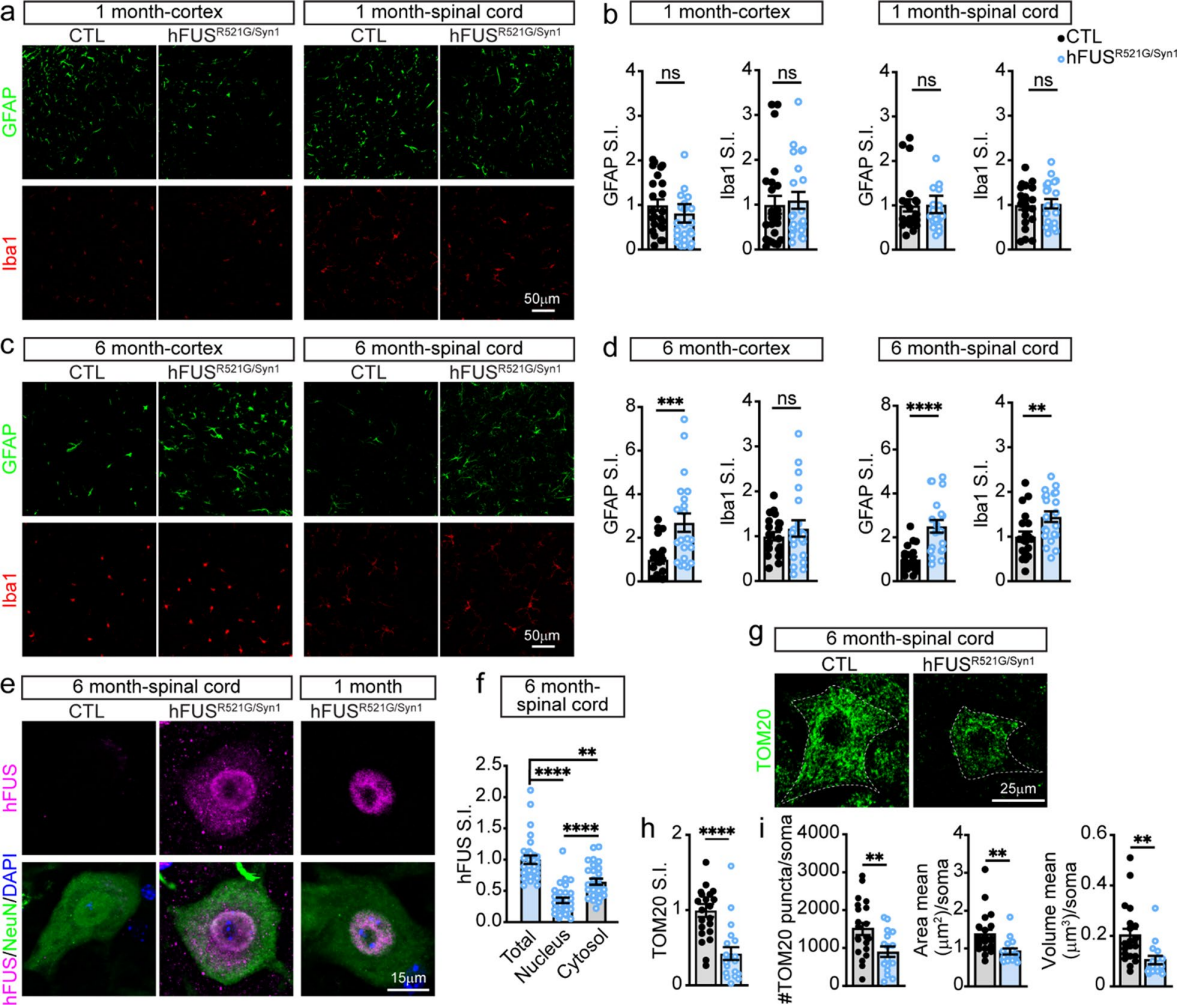
Neuron-specific expression of FUSR521G promotes age-dependent neuropathological features of ALS/FTD. The brain and spinal cord of **a** 1-month-old and **c** 6-months-old CTL and hFUS^R521G/Syn1^ mice stained with anti-GFAP (astrocyte marker) and anti-Iba1 (microglia marker). Signal intensity (S.I) for GFAP and Iba1 show **b** no significant changes in 1-month-old hFUS^R521G/Syn1^ mice and **d** significant activation of astrocytes and microglia in 6-months-old FUS transgenic mice compared to CTL mice. **e**,** f** Spinal cord staining with anti-hFUS, anti-NeuN and DAPI show mislocalisation of hFUS in 6-months-old hFUS^R521G/Syn1^ mice. **g** Spinal cord staining with the mitochondrial marker, TOM20. Quantification of TOM20 shows a significant decrease in **h** S.I. and **i** number of puncta, area and volume in 6-months-old hFUS^R521G/Syn1^ mice. Values from each group are expressed as mean ± SEM. Statistics uses an unpaired Student’s *t*-test for comparison between two groups and a one-way ANOVA for multiple group comparisons (n = 3–4 mice/group). **p < 0.01, ***p < 0.005 ****p < 0.001, and not significant (ns)

Glial activation, protein mislocalisation and aggregation, and mitochondrial impairments are neuropathological features of ALS/FTD implicated in the manifestation of the disease [12]. It is unclear how these pathological features relate to behavioral deficits, and changes in neuromorphology and synapses in hFUS^R521G/Syn1^ mice. 1- and 6-month-old hFUS^R521G/Syn1^ mice were assessed for astrocyte and microglial activation. There was no evidence of glial activation in the brains and spinal cords of 1-month-old hFUS^R521G/Syn1^ mice (Fig. 4a, b). 6-months-old hFUS^R521G/Syn1^ mice had significant astrogliosis in the cortex and spinal cord (Fig. 4c, d) and significant microgliosis in the spinal cord (Fig. 4c, d). However, activated microglia was not observed in the cortex of 6-months-old hFUS^R521G/Syn1^ mice (Fig. 4c, d). The reason for the lack of microglial activation in the cortex of 6-months-old hFUS^R521G/Syn1^ mice is not clear but may reflect the reported role of astrocytes in initiating the activation of microglia [79–81].

The 1- and 6-month-old hFUS^R521G/Syn1^ mice were also examined for changes in FUSR521G localisation and mitochondrial abnormalities. The subcellular distribution of FUSR521G remained prominently nuclear in 1-month-old hFUS^R521G/Syn1^ mice (Figs. 1e and 4e). In 6-months-old hFUS^R521G/Syn1^ mice there was an observable increase in the distribution of FUSR521G to the cytoplasm of spinal motor neurons at this age (Fig. 4e, f). Consistent with the behavioral, cellular and molecular changes observed in 6-months-old hFUS^R521G/Syn1^ mice, there was a significant reduction in the neuronal expression of TOM20 (Fig. 4g–i), a subunit of the TOM complex responsible for protein import across the outer mitochondrial membrane, whose expression correlates with mitochondrial activity [66].

Collectively, these findings indicate that in addition to the cell-autonomous effects of neuron-specific expression of FUSR521G, the changes that occur in motor neurons also promote non-cell autonomous events that involve glial activation. Moreover, these findings demonstrate that behavioral severity of 6-months-old hFUS^R521G/Syn1^ mice corresponds with a wider range of neuropathological features observed in humans with familial ALS-FUS or FTD-FUS [9–11].

### Inhibition of the canonical NF-κB pathway attenuates neural inflammation and restores neuromorphology and behavior deficits

**Fig. 5:**
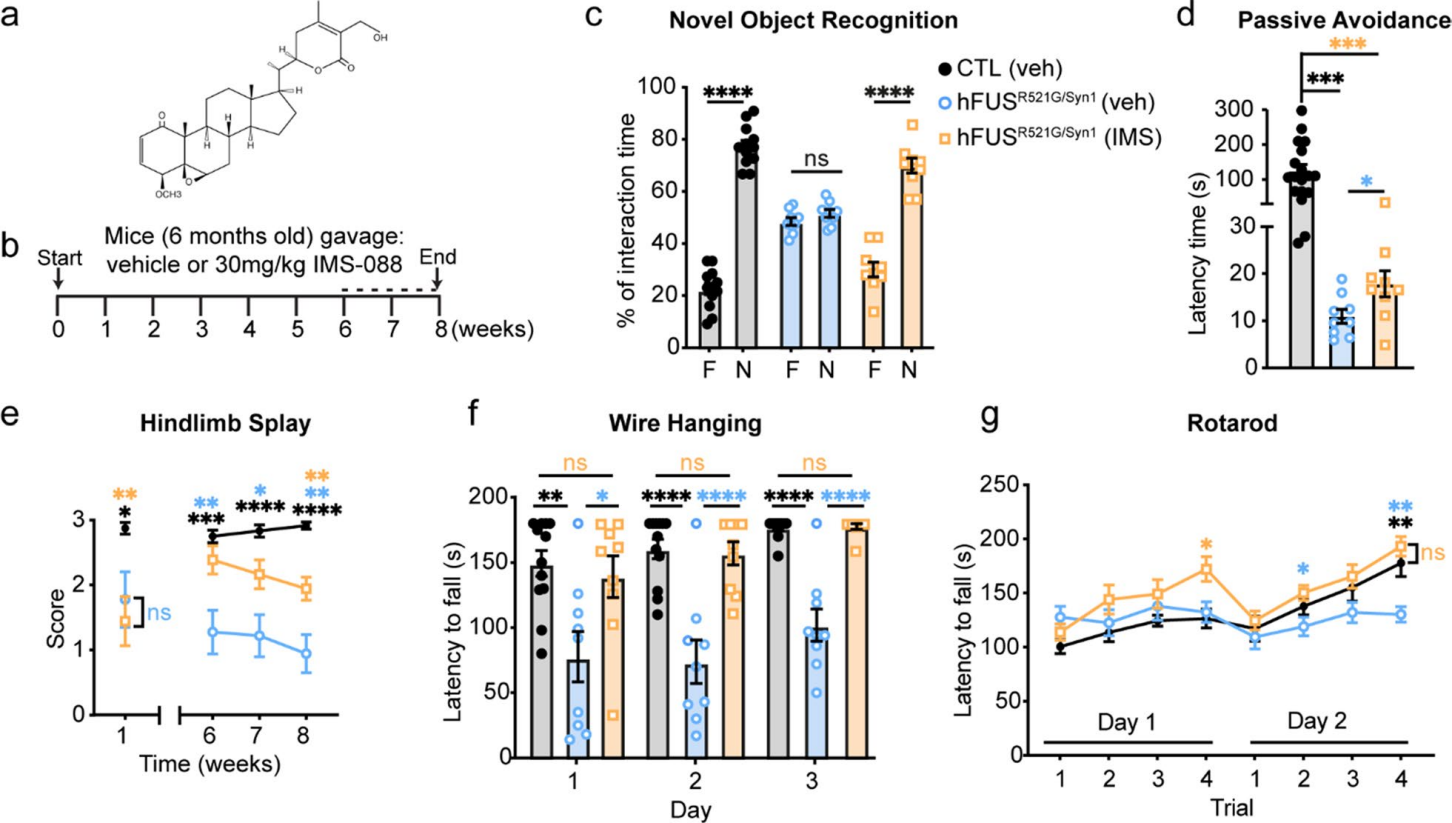
hFUS^R521G/Syn1^ mice treated with IMS-088 improves cognitive and motor function. **a** Chemical structure of IMS-088. **b** Schematic of treatment and behavior analysis of mice. Dotted line indicates when behavior tests were performed. Treatment of hFUS^R521G/Syn1^ mice with IMS-088 (IMS) improves their cognitive and motor performance compared with vehicle (veh) treated littermate control (CTL) and hFUS^R521G/Syn1^ mice. hFUS^R521G/Syn1^ mice treated with IMS-088 display **c** significant improvement in novel object recognition and **d** modest improvement in the passive avoidance test. FUS transgenic mice treated with IMS-088 display improved motor function as determined by **e** hindlimb splay, **f** wire hanging, and **g** rotarod tests. Values from each group are expressed as mean ± SEM. Statistics uses an unpaired Student’s *t*-test for comparison between two groups and a one-way ANOVA for multiple group comparisons (n = 9 hFUS^R521G/Syn1^(veh), n = 9 hFUS^R521G/Syn1^(IMS) and n = 12 CTL (veh) mice/group). *p < 0.05, **p < 0.01, ***p < 0.005 ****p < 0.001, and not significant (ns). For multiple group comparisons: black (*)/ns: CTL(veh) versus hFUS^R521G/Syn1^(veh), orange (*)/ns: CTL (veh) versus hFUS^R521G/Syn1^(IMS) and blue (*)/ns: hFUS^R521G/Syn1^(veh) versus hFUS^R521G/Syn1^(IMS)

The NF-κB pathway promotes neuroinflammation and the activation of astrocytes and microglia in ALS/FTD [82]. Sustained glial activation directly damages motor neurons and promotes disease progression [83–85]. In ALS/FTD models of disease, inhibition of the canonical NF-κB pathway with Withaferin A, an inhibitor of nuclear factor kappa-B kinase subunit gamma (IKK-γ) (also known as NEMO) [86–88] or IMS-088, an analog of Withaferin A, provides therapeutic benefits [69–71]. To test the effects of inhibiting NF-κB in hFUS^R521G/Syn1^ mice, IMS-088 or vehicle was administered to 6-months-old hFUS^R521G/Syn1^ mice daily for a duration of 8 weeks (Fig. 5a, b). 6-months-old mice were selected for IMS-088 administration based on the emerging motor decline and glial activation observed in these animals (Figs. 2 and 4). Vehicle-treated littermate control mice were also included in this analysis to establish the level of recovery in drug-treated mice. IMS-088-treated hFUS^R521G/Syn1^ mice showed significant cognitive improvement, as measured by the novel object recognition test, when compared to vehicle-treated mice (Fig. 5c). However, these mice only showed partial recovery of cognition, as measured by the passive avoidance test (Fig. 5d). Significant motor improvements were also observed in IMS-088-treated hFUS^R521G/Syn1^ mice as determined by hindlimb splay, wire hanging and rotarod tests, when compared to vehicle-treated mice (Fig. 5e–g).

**Fig. 6:**
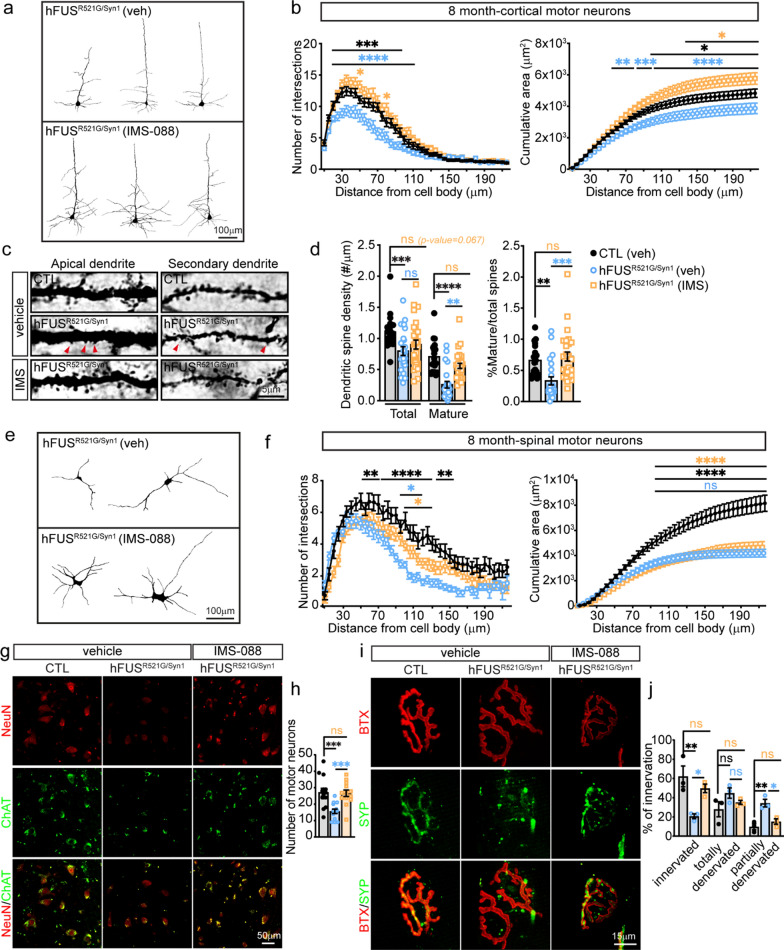
IMS-088 treatment of hFUS^R521G/Syn1^ mice restores neuromorphology and synapses. **a** Neurolucida tracing of cortical motor neuron dendrites from vehicle and IMS-088 treated hFUS^R521G/Syn1^ mice. **b** Sholl analysis shows a significant increase in the dendritic intersections and cumulative area of dendrites in cortical motor neurons of hFUS^R521G/Syn1^ mice treated with IMS-088 (IMS) compared to vehicle (veh) treated mice. **c** Golgi images of dendritic spines from layer IV–V neurons in the motor cortex from treated mice and **d** their quantification. There is a significant increase in the dendritic spine density of mature spines of hFUS^R521G/Syn1^ mice treated with IMS-088. **e** Neurolucida tracing of the dendrites of spinal motor neurons from hFUS^R521G/Syn1^ mice treated mice. **f** Sholl analysis shows some improvement in dendritic intersections of IMS-088 treated mice, but no improvement in cumulative area of dendrites in spinal motor neurons. **g** The spinal cord of CTL and hFUS^R521G/Syn1^ mice stained with anti-ChAT (motor neuron marker) and anti-NeuN. **h** Quantification of spinal motor neuron co-stained with ChAT and NeuN. **i** Neuromuscular junctions from gastrocnemius stained with α-bungarotoxin-647 (BTX) and anti-synaptophysin (SYP). **j** Quantification of neuromuscular junctions co-stained with BTX and SYP. Values from each group are expressed as mean ± SEM. Statistics uses two-way repeat measures ANOVA for Sholl analysis and one-way ANOVA for multiple group comparisons (n = 3–4 mice/group). *p < 0.05, **p < 0.01, ***p < 0.005 ****p < 0.001, and not significant (ns). For multiple group comparisons: black (*)/ns: CTL(veh) versus hFUS^R521G/Syn1^(veh), orange (*)/ns: CTL (veh) versus hFUS^R521G/Syn1^(IMS) and blue (*)/ns: hFUS^R521G/Syn1^(veh) versus hFUS^R521G/Syn1^(IMS)

The cortical and spinal motor neurons of IMS-088 and vehicle-treated mice were analysed for changes in dendritic branching and spines. The dendritic branching and cumulative area of cortical motor neurons from IMS-088-treated hFUS^R521G/Syn1^ mice were restored to the same level of dendritic complexity as vehicle-treated controls (Fig. 6a, b). The number of mature spines and dendritic spine densities of cortical motor neurons were also restored to the same levels as vehicle-treated controls (Fig. 6c, d). However, the total number of spines in IMS-088-treated hFUS^R521G/Syn1^ mice did not fully return to vehicle-treated control levels (Fig. 6d). The dendritic branching of spinal motor neurons of IMS-088-treated hFUS^R521G/Syn1^ mice showed modest improvements and no changes in cumulative area compared to vehicle-treated control mice (Fig. 6e, f). The cell bodies of spinal motor neurons and neuromuscular junctions were examined in IMS-088-treated hFUS^R521G/Syn1^ mice and found to be restored to similar levels as vehicle-treated controls (Fig. 6g–j), supporting the observed improvements in motor function these mice (Fig. 5e–g).

**Fig. 7:**
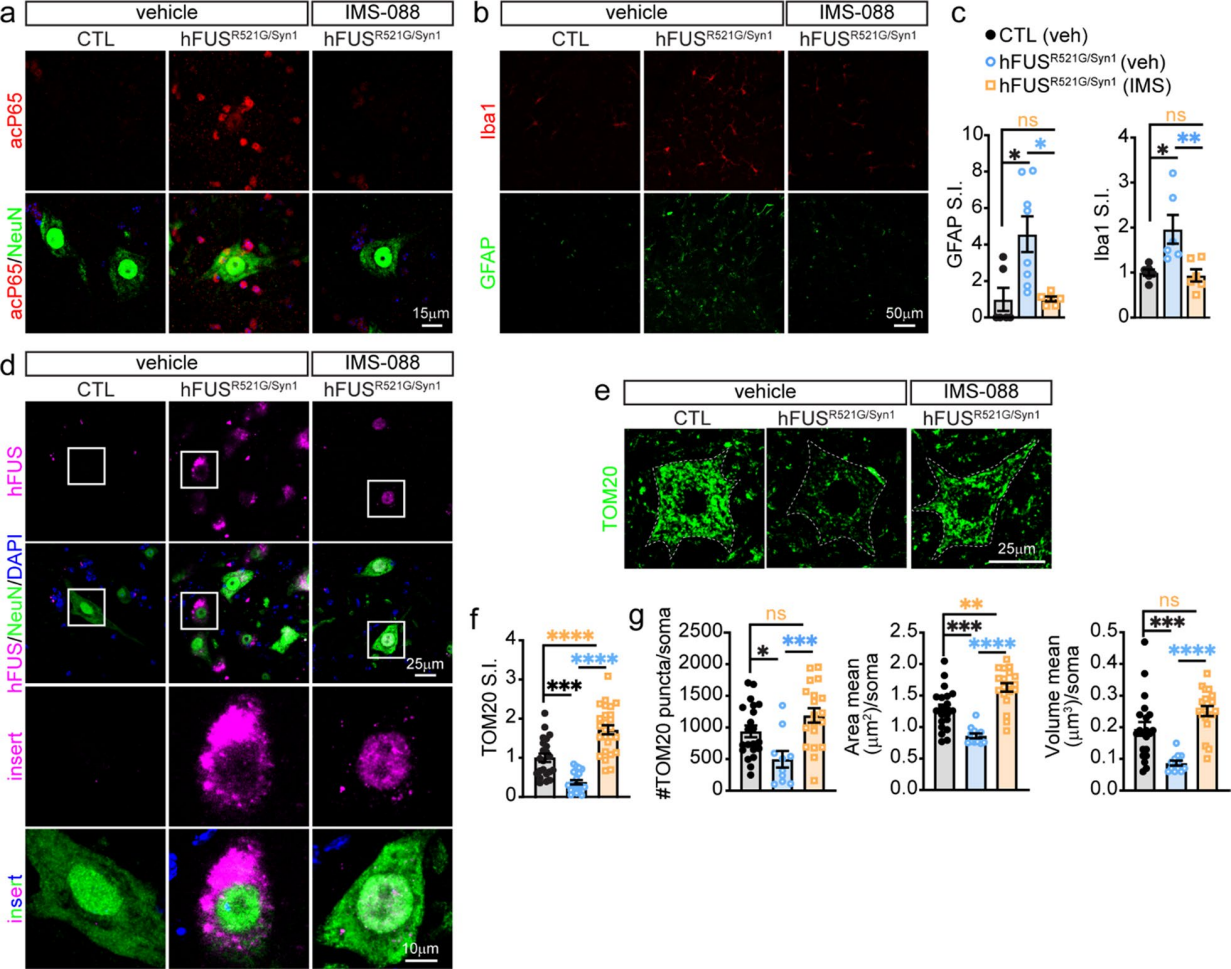
Inhibition of NF-κB with IMS-088 attenuates age-dependent pathological features in hFUS^R521G/Syn1^ mice. **a** The spinal cord of mice co-stained with anti-acetylated P65 (acP65) and anti-NeuN show that IMS-088 blocks the activation and nuclear localisation of acP65 in neurons and non-neuronal cells of treated hFUS^R521G/Syn1^ mice. **b** Spinal cord staining with anti-GFAP and anti-Iba1 in mice. **c** Signal intensity (S.I) for GFAP and Iba1 shows IMS-088 inhibits activation of astrocytes and microglial in treated FUS transgenic mice compared to vehicle treated mice. **d** Spinal cord staining with anti-hFUS, anti-NeuN and DAPI show IMS-088 restores nuclear distribution of hFUS in hFUS^R521G/Syn1^ mice. **e** Spinal cord staining with anti-TOM20. Quantification of TOM20 shows that IMS-088 restores **f** S.I. and **g** number of puncta, area and volume in 6-months-old FUS transgenic mice. Values from each group are expressed as mean ± SEM. Statistics uses a one-way ANOVA for multiple group comparisons (n = 3–4 mice/group). *p < 0.05, **p < 0.01, ***p < 0.005, ****p < 0.001, and not significant (ns)

The effect of IMS-088 on glial activation, FUS mislocalisation, and mitochondrial impairments were examined in hFUS^R521G/Syn1^ mice. Consistent with the reported actions of IMS-088, there was a decrease in the nuclear signal of acetylated P65, used as a marker for the activated form of P65 [89], in neurons and non-neuronal cells (Fig. 7a), and an attenuation of astrocyte and microglial activation in hFUS^R521G/Syn1^ treated mice (Fig. 7b, c and Additional file 1: Fig. S7). IMS-088-treated hFUS^R521G/Syn1^ mice had a decrease in cytoplasmic mislocalisation of FUSR521G in spinal motor neurons (Fig. 7d) as well as restored TOM20 expression in neurons when compared to vehicle-treated controls (Fig. 7e–g). Collectively, these results demonstrate that inhibiting inflammatory signals through the canonical NF-κB pathway has a beneficial impact on motor neuron dendritic and synaptic structures, which in turn improve the motor deficits in hFUS^R521G/Syn1^ mice.

### Inhibition of the canonical NF-κB pathway restores dendritic mitochondrial stasis

**Fig. 8:**
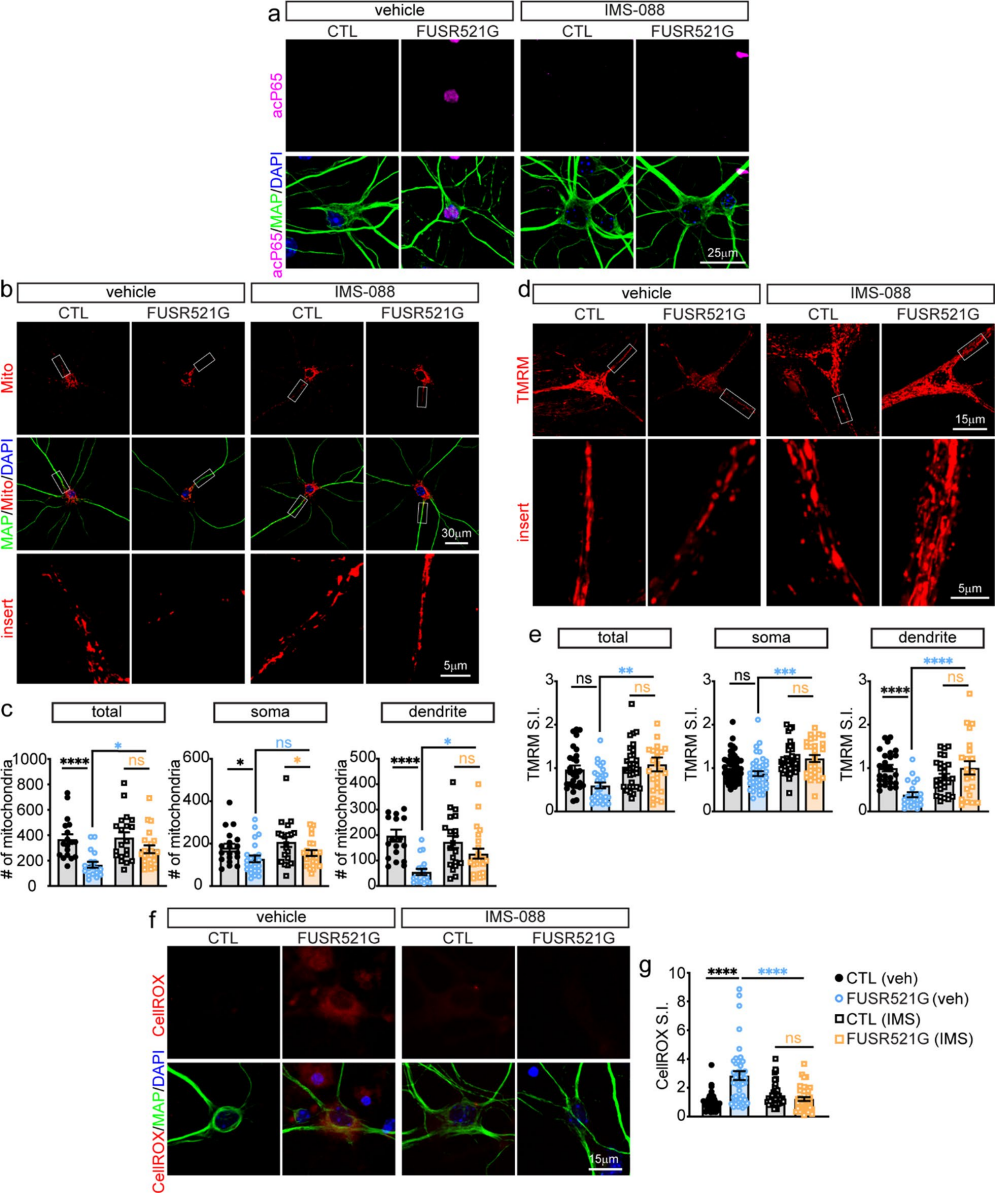
Inhibition of NF-κB with IMS-088 restores dendritic mitochondrial stasis in neurons expressing FUSR521G. **a** Primary cortical neurons cultured from hFUS^R521G/Meox^ (FUSR521G) or littermate control (CTL) mice treated with vehicle (veh) or IMS-088 co-stained with anti-acetylated P65 (acP65), anti-MAP2 (dendritic marker) and DAPI show IMS-088 blocks the activation and nuclear localisation of acP65 in neurons expressing FUSR521G. **b** Neuron cultures co-stained with Mitotracker, anti-MAP2 and DAPI. **c** Treatment with IMS-088 promotes an increase in the number of mitochondria within the dendrites of neurons expressing FUSR521G. **d** Live images of neurons stained with tetramethylrhodamine, methyl ester (TMRM). **e** Treatment with IMS-088 promotes an increase in the number of functional mitochondria within the dendrites of neurons expressing FUSR521G. **f** Neuron cultures co-stained with CellROX, anti-MAP2 and DAPI. **g** Treatment with IMS-088 blocks the production of reactive oxygen species in neurons expressing FUSR521G. Values from each group are expressed as mean ± SEM. Statistics uses a one-way ANOVA for multiple group comparisons (n = 3–4 biological replicates/group). *p < 0.05, **p < 0.01, ****p < 0.001, and not significant (ns)

In ALS/FTD, changes in neuromorphology and synaptic loss have been attributed to defects in mitochondrial function and trafficking to dendrites and synapses [63, 90–93]. IMS-088 treatment of hFUS^R521G/Syn1^ mice results in inhibition of NF-κB along with the restoration of TOM20 mitochondrial expression and increased dendritic branching and synapses of motor neurons (Figs. 6 and 7). However, it is unclear whether IMS-088 treatment restores the function of mitochondria in our model. To address this question, primary cortical neurons were cultured from germline hFUS^R521G/Meox2Cre^ transgenic and littermate control mice [42] and treated with vehicle or IMS-088. Similar to the observations made in vivo, nuclear acetylated P65 was present in FUSR521G expressing neurons and was attenuated with IMS-088 treatment (Fig. 8a). The total mitochondrial numbers were examined in these cultures and found to be significantly reduced in FUSR521G expressing neurons when compared with control neurons (Fig. 8b, c). Mitochondrial numbers in the soma and dendrites were analysed separately and found to be significantly reduced in both cellular compartments, but more prominently reduced in the dendrites of FUSR521G expressing neurons (Fig. 8b, c). In response to IMS-088 treatments, the total number and distribution of mitochondria in the dendrites of FUSR521G expressing neurons were nearly restored to the same levels as control neurons (Fig. 8b, c).

We reasoned that restoring the dendritic mitochondria in FUSR521G expressing neurons could also reflect changes in mitochondrial activity. The mitochondrial activity was assessed in FUSR521G expressing neurons using tetramethylrhodamine, methyl ester (TMRM), a fluorescent dye taken-up by active mitochondria. FUSR521G expressing neurons had a significant reduction in dendritic mitochondria activity and no differences in the total or soma mitochondrial activity when compared with control neurons (Fig. 8d, e). Treatment of FUSR521G expressing neurons with IMS-088 resulted in the restoration of mitochondrial activity to the same levels observed in control neurons (Fig. 8d, e). Consistent with the restoration of mitochondrial activity due to IMS-088 treatments (Fig. 8b–e), FUSR521G expressing neurons treated with IMS-088 also had a significant reduction in the level of reactive oxygen species when compared to control cultures (Fig. 8f, g). Taken together, our findings indicate that inhibition of the pro-inflammatory canonical NF-κB pathway using IMS-088 lowers oxidative stress and has a positive effect on mitochondrial stasis in neurons.

## Discussion

Dendritic attrition of motor neurons and loss of synaptic connectivity within motor neuron networks have emerged as early and common pathological features of ALS and FTD. In order to gain a deeper understanding of how changes in neuromorphology and synaptic loss relate to disease progression in ALS/FTD, we generated a neuron-specific FUS-transgenic mouse, hFUS^R521G/Syn1^, and longitudinally assessed these pathological features and their relationship to other ALS/FTD-associated phenotypes. Here we show that neuron-restricted expression of the ALS-linked FUSR521G variant in mice causes early cell-autonomous changes in the dendritic branching of cortical motor neurons that precede motor deficits in hFUS^R521G/Syn1^ mice. Age-dependent defects in motor function occur in hFUS^R521G/Syn1^ mice and coincide with more prominent changes in neuromorphology of cortical and spinal motor neurons and synaptic loss. Importantly, we found that glial activation, FUS mislocalisation and defects in mitochondria occur later in symptomatic adult mice. These findings demonstrate that expression of FUSR521G in neurons promotes a sequence of cell-autonomous changes that initiate ALS-associated phenotypes, including the non-cell autonomous activation of glia, which in turn contributes to the worsening of disease phenotypes. To attenuate motor neuron damage potentially caused by sustained glial activation, we treated hFUS^R521G/Syn1^ mice with IMS-088, an inhibitor of the canonical NF-κB pathway [69–71]. We show that inhibition of the NF-κB pathway with IMS-088 improves cognitive and motor function, increases dendritic branching of motor neurons and restores synapses in hFUS^R521G/Syn1^ mice. Investigation into the mechanism of action by IMS-088 in vitro show that inhibition of NF-κB in FUSR521G expressing neurons reduces oxidative stress and positively affects mitochondrial stasis. Collectively, our findings demonstrate that the ALS-linked FUSR521G mutant causes cell-autonomous defects that contribute to ALS/FTD-associated pathologies, and that targeting the canonical NF-κB pathway is a therapeutic strategy for attenuating gliosis and maintaining corticospinal connectivity in ALS/FTD.

We also identified that neuron-restricted expression of FUSR521G caused early cognitive defects in hFUS^R521G/Syn1^ mice, which reflects nearly 50% of ALS cases that have either cognitive (ALSci) or behavioral (ALSbi) impairment, or both (ALScbi), in addition to motor impairment [1–3]. Notably, individuals with ALSbi and ALScbi have significantly higher cortical motor neuron involvement [94, 95]. Behavioral changes and cognitive abnormalities in ALS correspond with degeneration of the frontal and temporal lobes [1], which are similarly affected in FTD-subtypes [96, 97]. The cognitive abnormalities in ALS and shared clinical features within the spectrum of FTD, suggest a common pathophysiological mechanism to account for cognitive defects in these diseases. However, the sequence of events that promote motor deficits and behavioral and cognitive impairments in ALS are unclear. ALS is proposed to begin in the cerebral neocortex regions where it progressively affects spinal motor neurons and extra-motor brain regions [76, 77]. Here we show that expression of FUSR521G in neurons causes cognitive impairments that precede motor decline in hFUS^R521G/Syn1^ mice (Figs. 1 and 2). We report early cell-autonomous changes in the dendritic branching of cortical motor neurons in juvenile hFUS^R521G/Syn1^ mice, followed by morphological changes in spinal motor neurons, synaptic loss and other pathological features of ALS in aged mice (Figs. 3 and 4). Based on the early cognitive defects in our model and the morphological changes in cortical motor neurons, our findings point to other neuron populations contributing to cognitive defects in our model. In another transgenic model expressing FUSR514G under control of the prion promotor, age-dependent cognitive defects along with decreased dendritic spine density and long-term potentiation in the hippocampus are reported [63]. Thus, future work in hFUS^R521G/Syn1^ mice will need to examine the other neuron populations implicated in cognitive function to understand the cognitive impairments in our model. Collectively, our findings suggest the sequence of events that lead to loss of corticospinal connectivity in hFUS^R521G/Syn1^ mice originate in the brain due to selective vulnerability of neurons to FUSR521G. The collective body of evidence suggests that cognitive impairments in ALS can precede motor dysfunction, thus providing a strong rational for more rigorous assessment of cognitive and behavioral impairments in ALS patients and in models of disease.

The pathology associated with ALS/FTD is mediated by multiple different cell-types [67, 68]. However, the contribution of these cell-types to changes in neuromorphology and synapses are not well understood. While changes in neuromorphology and synaptic loss have been reported in mouse models of ALS-FUS [42, 61, 63], other pathological features of ALS/FTD such as glial activation and protein aggregation coincide with these changes, making it difficult to determine the cell-type contributions to these defects. We examined hFUS^R521G/Syn1^ mice in the early stages of disease and found that neuron-restricted expression of FUSR521G caused cell-autonomous changes to the neuromorphology of cortical motor neurons in the absence of FUSR521G aggregation (Fig. 4), suggesting that the mutant is sufficient to promote these changes. Our findings are consistent with reports that ALS-linked FUS variants, but not human FUS wild-type, cause dendritic attrition and loss of synapses [42, 62, 64]. Indeed, FUS has important functions in dendritic growth, maturation of spines and synaptic transmission [50, 52, 98]. Deletion of FUS in primary neurons causes altered dendritic branching and immature dendritic spines [99], down-regulation of AMPA receptor surface expression and reduced miniature excitatory postsynaptic potential (EPSC) amplitudes [52]. Dysregulation of FUS also corresponds with an accumulation of RNA in the dendrites [99] and synapses [100], consistent with the reported functions of FUS in mRNA transport, stability and translation regulation [49–54]. Future studies will need to examine the molecular changes that occur within motor neurons expressing ALS-linked FUS variants to better understand the effects of FUS on motor neuron structure and synaptic integrity.

In addition to the cell-autonomous changes caused by FUSR521G in neurons, we also found that the activation of astrocytes and microglia coincided with worsening behavior and pathological phenotypes in these mice (Figs. 2, 3 and 4). These findings suggest that neuronal defects caused by FUSR521G are subsequently promoting the activation of glia, which have been shown to act in a non-cell autonomous manner to cause ALS/FTD pathology [67, 68]. Notability, activation of microglia was not observed in the cortex of 6-months-old mice (Fig. 4) but observed later in 8-months-old mice (Additional file 1: Fig. S7). Although the implications of this are not clear, other animal models of ALS have report that activation of astrocytes can precede microglia activation and are involved in their induction [80, 81]. While the non-cell autonomous contributions of astrocytes and microglia to changes in neuromorphology and synapses were not examined in our model, studies show that these cell-types have important roles in the maintenance of these structures [101–103]. Indeed, ALS-linked FUS variants have been shown to affect glial function. The expression of the ALS-linked FUSR521G mutant in astrocytes induces motor neuron death in vitro through release of the pro-inflammatory cytokine tumor necrosis factor-alpha (TNFα) [104]. Additionally, induced pluripotent stem cell (iPSC)-derived microglia expressing the ALS-linked FUSP525L mutant have altered transcriptome profiles including genes involved in chemoreceptor-activated calcium signalling, alterations which are predicted to affect their phagocytic roles during neuroinflammation [105]. It is therefore likely that the combination of both cell-autonomous and non-cell autonomous effects of ALS-linked FUS variants in different cell-types contributes to the severity of ALS/FTD pathology that is observed in humans. In the context of our hFUS^R521G/Syn1^ mice, neuron-restricted expression of FUSR521G produces less severe phenotypes when compared with global expression models of FUSR521G, which have more severe phenotypes [42]. This suggests that other cell-types are also affected by FUS mutations and that they too contribute to ALS-associated phenotypes. Further investigation into the role of FUS expression in glial cells and their contribution to changes in neuromorphology and synaptic loss pathologies will provide additional information on the non-cell autonomous effects of these cell-types on these early pathological features of ALS/FTD.

Neuroinflammation and mitochondrial dysfunction are associated with ALS and FTD pathology [106]. Consistent with these findings, our FUSR521G models have increased activation of the pro-inflammatory NF-κB pathway and changes in mitochondrial numbers, activity, and distribution within dendrites (Figs. 7 and 8). Using IMS-088 to inhibit the canonical NF-κB pathway, we demonstrate that reducing pro-inflammatory signals has an overall therapeutic effect on the reversal of ALS/FTD associated phenotypes observed in our models (Figs. 5, 6, 7 and 8). We find that IMS-088 treatment improves cognitive and motor phenotypes in hFUS^R521G/Syn1^ mice and restores dendritic branches and synapses (Figs. 5 and 6). Additionally, treatment with IMS-088 attenuates inflammation, restores FUS mislocalisation and mitochondrial defects in hFUS^R521G/Syn1^ mice (Fig. 7). Neuroinflammation is shown to promote structural defects in neurons including dendritic attrition and synaptic loss [107–109]. Moreover, sustained activation of pro-inflammatory NF-κB pathways negatively impact mitochondrial number, cellular distribution and function [110, 111]. The maintenance of mitochondria function and distribution within the neuron is critical for maintaining dendritic structures and synaptic integrity [90, 91, 112, 113]. Our findings indicate that inhibition of NF-κB restores mitochondrial status and promotes phenotypic improvements in our models of ALS-FUS. This important relationship between the NF-κB pathway and mitochondrial function provides new insights into the pathways and mechanisms involved in the defects in neuromorphology and synaptic loss associated with ALS/FTD. Moreover, these findings may have more broad implications for neurodegenerative diseases that share similar pathological features.

ALS-linked FUS variants are implicated in several pathological changes at the cellular level including mitochondrial dysfunction and pro-inflammatory signals [12]. We show both mitochondrial dysfunction and pro-inflammatory signals are present in our models (Figs. 7 and 8), but the role of FUSR521G in promoting these changes was not investigated in our study. However, ALS-linked FUS variants are reported to cause defects in mitochondrial function through aberrant interactions with mRNA or protein interactions that lead to global changes in protein synthesis [114–116]. Alterations in mitochondrial abundance and function occur in ALS/FTD-FUS patients and other FUS models [114, 117, 118]. Additionally, FUS is implicated in pro-inflammatory signals. FUS acts as a coactivator of NF-κB in response to pro-inflammatory cytokines [119]. Cells treated with activators of pro-inflammatory pathways, have a corresponding increase in FUS mRNA stability and protein expression [120]. Moreover, expression of ALS-FUS variants or overexpression of wild-type FUS in glia have increased sensitivity to pro-inflammatory signals [104, 121]. It is therefore possible and likely that FUS misregulation causes systemic defects at various cellular levels including those involving mitochondrial function and pro-inflammatory signals. Indeed, neuroinflammation and mitochondrial dysfunction have complex connections that promote disease pathology. Damaged mitochondria release factors that are recognized by immune receptors of microglia and promote neuroinflammation [122, 123]. Conversely, inflammatory factors released by activated glia can trigger an intracellular cascade that can affect mitochondrial metabolism and function [110, 111]. Our findings show inhibiting the NF-κB pathway is sufficient to restore aspects of cellular homeostasis and promote phenotypic recovery in our FUSR521G models, suggesting that pro-inflammatory signals are implicated ALS-associated phenotypes. It is unclear from our FUSR521G models, which occurs first: mitochondrial dysfunction or neuroinflammation, or whether they occur in parallel. Future studies will examine the mechanistic actions of inhibiting the NF-κB pathway on FUS function and the dynamic interplay between pro-inflammatory signals and mitochondrial function in disease models of ALS/FTD.

### Supplementary Information

**Additional file 1**. **Supplementary methods and figures:** Methods. Material and methods for supplemental data. Figure legends. Figure legends for supplemental figures. **Fig. S1.** hFUS co-staining with markers for astrocytes or microglia. **Fig. S2.** Excision and expression analysis of hFUS in the brain and spinal cord of hFUS^R521/Syn1^ mice. **Fig. S3.** hFUS^R521/Syn1^ mice present persistent cognitive impairment, along with progressive, age-dependent motor dysfunction. **Fig. S4.** No sex-dependent differences are observed in hFUShFUS^R521/Syn1^ mice. **Fig. S5.** Progressive loss of motor neurons and NMJ denervation in aged hFUS^R521/Syn1^ mice. **Fig. S6.** No defects in spinal motor neurons and NMJ in 6-month-old hFUS^R521/Syn1^ mice. **Fig. S7.** IMS-088 treatment attenuates glial activation in the cortex of hFUS^R521/Syn1^ mice.

## References

[1] Lomen-Hoerth C, Anderson T, Miller B (2002). The overlap of amyotrophic lateral sclerosis and frontotemporal dementia. Neurology.

[2] Strong MJ (2017). Amyotrophic lateral sclerosis - frontotemporal spectrum disorder (ALS-FTSD): revised diagnostic criteria. Amyotroph Lateral Scler Frontotemporal Degener.

[3] Benbrika S (2019). Cognitive, emotional and psychological manifestations in amyotrophic lateral sclerosis at baseline and overtime: a review. Front Neurosci.

[4] Lipton AM, White CL, Bigio EH (2004). Frontotemporal lobar degeneration with motor neuron disease-type inclusions predominates in 76 cases of frontotemporal degeneration. Acta Neuropathol.

[5] Renton AE (2011). A hexanucleotide repeat expansion in C9ORF72 is the cause of chromosome 9p21-linked ALS-FTD. Neuron.

[6] DeJesus-Hernandez M (2011). Expanded GGGGCC hexanucleotide repeat in noncoding region of C9ORF72 causes chromosome 9p-linked FTD and ALS. Neuron.

[7] Neumann M (2006). Ubiquitinated TDP-43 in frontotemporal lobar degeneration and amyotrophic lateral sclerosis. Science.

[8] Arai T (2006). TDP-43 is a component of ubiquitin-positive tau-negative inclusions in frontotemporal lobar degeneration and amyotrophic lateral sclerosis. Biochem Biophys Res Commun.

[9] Vance C (2009). Mutations in FUS, an RNA processing protein, cause familial amyotrophic lateral sclerosis type 6. Science.

[10] Kwiatkowski TJ (2009). Mutations in the FUS/TLS gene on chromosome 16 cause familial amyotrophic lateral sclerosis. Science.

[11] Neumann M (2009). A new subtype of frontotemporal lobar degeneration with FUS pathology. Brain.

[12] Gelon PA, Dutchak PA, Sephton CF (2022). Synaptic dysfunction in ALS and FTD: anatomical and molecular changes provide insights into mechanisms of disease. Front Mol Neurosci.

[13] Van Langenhove T (2010). Genetic contribution of FUS to frontotemporal lobar degeneration. Neurology.

[14] Huey ED (2012). FUS and TDP43 genetic variability in FTD and CBS. Neurobiol Aging.

[15] Munoz DG (2009). FUS pathology in basophilic inclusion body disease. Acta Neuropathol.

[16] Suzuki N (2012). FUS/TLS-immunoreactive neuronal and glial cell inclusions increase with disease duration in familial amyotrophic lateral sclerosis with an R521C FUS/TLS mutation. J Neuropathol Exp Neurol.

[17] Svetoni F, Frisone P, Paronetto MP (2016). Role of FET proteins in neurodegenerative disorders. RNA Biol.

[18] Nicolas G (2022). A postzygotic de novo NCDN mutation identified in a sporadic FTLD patient results in neurochondrin haploinsufficiency and altered FUS granule dynamics. Acta Neuropathol Commun.

[19] Seelaar H (2010). Frequency of ubiquitin and FUS-positive, TDP-43-negative frontotemporal lobar degeneration. J Neurol.

[20] Hammer RP, Tomiyasu U, Scheibel AB (1979). Degeneration of the human Betz cell due to amyotrophic lateral sclerosis. Exp Neurol.

[21] Horoupian DS (1984). Dementia and motor neuron disease: morphometric, biochemical, and Golgi studies. Ann Neurol.

[22] Genc B (2017). Apical dendrite degeneration, a novel cellular pathology for Betz cells in ALS. Sci Rep.

[23] Ferrer I (1991). Dementia of frontal lobe type and motor neuron disease. A Golgi study of the frontal cortex. J Neurol Neurosurg Psychiatry.

[24] Kato T, Hirano A, Donnenfeld H (1987). A Golgi study of the large anterior horn cells of the lumbar cords in normal spinal cords and in amyotrophic lateral sclerosis. Acta Neuropathol.

[25] Henstridge CM (2018). Synapse loss in the prefrontal cortex is associated with cognitive decline in amyotrophic lateral sclerosis. Acta Neuropathol.

[26] Sasaki S, Maruyama S (1994). Decreased synaptophysin immunoreactivity of the anterior horns in motor neuron disease. Acta Neuropathol.

[27] Liu X, Erikson C, Brun A (1996). Cortical synaptic changes and gliosis in normal aging, Alzheimer’s disease and frontal lobe degeneration. Dementia.

[28] Ferrer I (1999). Neurons and their dendrites in frontotemporal dementia. Dement Geriatr Cogn Disord.

[29] Lipton AM (2001). Contribution of asymmetric synapse loss to lateralizing clinical deficits in frontotemporal dementias. Arch Neurol.

[30] Brun A, Liu X, Erikson C (1995). Synapse loss and gliosis in the molecular layer of the cerebral cortex in Alzheimer’s disease and in frontal lobe degeneration. Neurodegeneration.

[31] Laszlo ZI (2022). Synaptic proteomics reveal distinct molecular signatures of cognitive change and C9ORF72 repeat expansion in the human ALS cortex. Acta Neuropathol Commun.

[32] Umoh ME (2018). A proteomic network approach across the ALS-FTD disease spectrum resolves clinical phenotypes and genetic vulnerability in human brain. EMBO Mol Med.

[33] Iridoy MO (2018). Neuroanatomical quantitative proteomics reveals common pathogenic biological routes between amyotrophic lateral sclerosis (ALS) and frontotemporal dementia (FTD). Int J Mol Sci.

[34] Martins-de-Souza D (2012). Proteomic analysis identifies dysfunction in cellular transport, energy, and protein metabolism in different brain regions of atypical frontotemporal lobar degeneration. J Proteome Res.

[35] Malpetti M (2021). Synaptic density in carriers of C9orf72 mutations: a [(11) C]UCB-J PET study. Ann Clin Transl Neurol.

[36] Lall D (2021). C9orf72 deficiency promotes microglial-mediated synaptic loss in aging and amyloid accumulation. Neuron.

[37] Wu LS (2019). Transcriptomopathies of pre- and post-symptomatic frontotemporal dementia-like mice with TDP-43 depletion in forebrain neurons. Acta Neuropathol Commun.

[38] Handley EE (2017). Synapse dysfunction of layer V pyramidal neurons precedes neurodegeneration in a mouse model of TDP-43 proteinopathies. Cereb Cortex.

[39] Dyer MS, Woodhouse A, Blizzard CA (2021). Cytoplasmic human TDP-43 mislocalization induces widespread dendritic spine loss in mouse upper motor neurons. Brain Sci.

[40] Dyer MS (2021). Mislocalisation of TDP-43 to the cytoplasm causes cortical hyperexcitability and reduced excitatory neurotransmission in the motor cortex. J Neurochem.

[41] Fogarty MJ (2016). Cortical synaptic and dendritic spine abnormalities in a presymptomatic TDP-43 model of amyotrophic lateral sclerosis. Sci Rep.

[42] Sephton CF (2014). Activity-dependent FUS dysregulation disrupts synaptic homeostasis. Proc Natl Acad Sci U S A.

[43] Rogelj B (2012). Widespread binding of FUS along nascent RNA regulates alternative splicing in the brain. Sci Rep.

[44] Ishigaki S (2012). Position-dependent FUS-RNA interactions regulate alternative splicing events and transcriptions. Sci Rep.

[45] Hoell JI (2011). RNA targets of wild-type and mutant FET family proteins. Nat Struct Mol Biol.

[46] Lagier-Tourenne C (2012). Divergent roles of ALS-linked proteins FUS/TLS and TDP-43 intersect in processing long pre-mRNAs. Nat Neurosci.

[47] Mastrocola AS (2013). The RNA-binding protein fused in sarcoma (FUS) functions downstream of poly(ADP-ribose) polymerase (PARP) in response to DNA damage. J Biol Chem.

[48] Schwartz JC (2012). FUS binds the CTD of RNA polymerase II and regulates its phosphorylation at Ser2. Genes Dev.

[49] Kapeli K (2016). Distinct and shared functions of ALS-associated proteins TDP-43, FUS and TAF15 revealed by multisystem analyses. Nat Commun.

[50] Fujii R, Takumi T (2005). TLS facilitates transport of mRNA encoding an actin-stabilizing protein to dendritic spines. J Cell Sci.

[51] Kamelgarn M (2018). ALS mutations of FUS suppress protein translation and disrupt the regulation of nonsense-mediated decay. Proc Natl Acad Sci U S A.

[52] Udagawa T (2015). FUS regulates AMPA receptor function and FTLD/ALS-associated behaviour via GluA1 mRNA stabilization. Nat Commun.

[53] Morlando M (2012). FUS stimulates microRNA biogenesis by facilitating co-transcriptional Drosha recruitment. EMBO J.

[54] Sevigny M (2020). FUS contributes to mTOR-dependent inhibition of translation. J Biol Chem.

[55] Vance C (2013). ALS mutant FUS disrupts nuclear localization and sequesters wild-type FUS within cytoplasmic stress granules. Hum Mol Genet.

[56] Dormann D (2010). ALS-associated fused in sarcoma (FUS) mutations disrupt Transportin-mediated nuclear import. EMBO J.

[57] Sephton CF, Yu G (2015). The function of RNA-binding proteins at the synapse: implications for neurodegeneration. Cell Mol Life Sci.

[58] Alhindi A, Boehm I, Chaytow H (2021) Small junction, big problems: neuromuscular junction pathology in mouse models of amyotrophic lateral sclerosis (ALS)*.* J Anat 10.1111/joa.13463 PMC9558162 34101196

[59] Shang Y, Huang EJ (2016). Mechanisms of FUS mutations in familial amyotrophic lateral sclerosis. Brain Res.

[60] Carey JL, Guo L (2022). Liquid-liquid phase separation of TDP-43 and FUS in physiology and pathology of neurodegenerative diseases. Front Mol Biosci.

[61] Qiu H (2014). ALS-associated mutation FUS-R521C causes DNA damage and RNA splicing defects. J Clin Invest.

[62] Tibshirani M (2017). Dysregulation of chromatin remodelling complexes in amyotrophic lateral sclerosis. Hum Mol Genet.

[63] Ho WY (2021). Dysfunction in nonsense-mediated decay, protein homeostasis, mitochondrial function, and brain connectivity in ALS-FUS mice with cognitive deficits. Acta Neuropathol Commun.

[64] Shiihashi G (2017). Dendritic homeostasis disruption in a novel frontotemporal dementia mouse model expressing cytoplasmic fused in sarcoma. EBioMedicine.

[65] Naumann M (2018). Impaired DNA damage response signaling by FUS-NLS mutations leads to neurodegeneration and FUS aggregate formation. Nat Commun.

[66] Wurm CA (2011). Nanoscale distribution of mitochondrial import receptor Tom20 is adjusted to cellular conditions and exhibits an inner-cellular gradient. Proc Natl Acad Sci U S A.

[67] Chen H (2018). Exploring the genetics and non-cell autonomous mechanisms underlying ALS/FTLD. Cell Death Differ.

[68] Ilieva H, Polymenidou M, Cleveland DW (2009). Non-cell autonomous toxicity in neurodegenerative disorders: ALS and beyond. J Cell Biol.

[69] Kumar S (2021). Induction of autophagy mitigates TDP-43 pathology and translational repression of neurofilament mRNAs in mouse models of ALS/FTD. Mol Neurodegener.

[70] Swarup V (2011). Deregulation of TDP-43 in amyotrophic lateral sclerosis triggers nuclear factor kappaB-mediated pathogenic pathways. J Exp Med.

[71] Patel P, Julien JP, Kriz J (2015). Early-stage treatment with Withaferin A reduces levels of misfolded superoxide dismutase 1 and extends lifespan in a mouse model of amyotrophic lateral sclerosis. Neurotherapeutics.

[72] Zhu Y (2001). Ablation of NF1 function in neurons induces abnormal development of cerebral cortex and reactive gliosis in the brain. Genes Dev.

[73] Leger M (2013). Object recognition test in mice. Nat Protoc.

[74] Dutta K (2017). Withania somnifera reverses transactive response DNA binding protein 43 proteinopathy in a mouse model of amyotrophic lateral sclerosis/frontotemporal lobar degeneration. Neurotherapeutics.

[75] Thammisetty SS (2021). Targeting TDP-43 pathology alleviates cognitive and motor deficits caused by chronic cerebral hypoperfusion. Neurotherapeutics.

[76] Braak H (2013). Amyotrophic lateral sclerosis–a model of corticofugal axonal spread. Nat Rev Neurol.

[77] Brettschneider J (2013). Stages of pTDP-43 pathology in amyotrophic lateral sclerosis. Ann Neurol.

[78] Dadon-Nachum M, Melamed E, Offen D (2011). The “dying-back” phenomenon of motor neurons in ALS. J Mol Neurosci.

[79] Tjalkens RB, Popichak KA, Kirkley KA (2017). Inflammatory Activation of Microglia and Astrocytes in Manganese Neurotoxicity. Adv Neurobiol.

[80] Yang WW (2011). Relationship between neuropathology and disease progression in the SOD1(G93A) ALS mouse. Exp Neurol.

[81] Jara JH (2019). MCP1-CCR2 and neuroinflammation in the ALS motor cortex with TDP-43 pathology. J Neuroinflamm.

[82] Lawrence T (2009). The nuclear factor NF-kappaB pathway in inflammation. Cold Spring Harb Perspect Biol.

[83] Alexianu ME, Kozovska M, Appel SH (2001). Immune reactivity in a mouse model of familial ALS correlates with disease progression. Neurology.

[84] Hall ED, Oostveen JA, Gurney ME (1998). Relationship of microglial and astrocytic activation to disease onset and progression in a transgenic model of familial ALS. Glia.

[85] Zhao W (2004). Activated microglia initiate motor neuron injury by a nitric oxide and glutamate-mediated mechanism. J Neuropathol Exp Neurol.

[86] Jackson SS (2015). Withaferin A disrupts ubiquitin-based NEMO reorganization induced by canonical NF-kappaB signaling. Exp Cell Res.

[87] Grover A (2010). Inhibition of the NEMO/IKKbeta association complex formation, a novel mechanism associated with the NF-kappaB activation suppression by Withania somnifera’s key metabolite withaferin A. BMC Genom.

[88] Hooper C (2014). Covalent modification of the NF-kappaB essential modulator (NEMO) by a chemical compound can regulate its ubiquitin binding properties in vitro. J Biol Chem.

[89] Pozzi S et al (2020) Monoclonal full-length antibody against TAR DNA binding protein 43 reduces related proteinopathy in neurons*.* JCI Insight 5(21) 10.1172/jci.insight.140420 PMC7710295 33021970

[90] Sasaki S, Iwata M (2007). Mitochondrial alterations in the spinal cord of patients with sporadic amyotrophic lateral sclerosis. J Neuropathol Exp Neurol.

[91] Okamoto K (1990). Axonal swellings in the corticospinal tracts in amyotrophic lateral sclerosis. Acta Neuropathol.

[92] Magrane J (2012). Mitochondrial dynamics and bioenergetic dysfunction is associated with synaptic alterations in mutant SOD1 motor neurons. J Neurosci.

[93] Sheng ZH, Cai Q (2012). Mitochondrial transport in neurons: impact on synaptic homeostasis and neurodegeneration. Nat Rev Neurosci.

[94] Maranzano A (2022). Upper motor neuron dysfunction is associated with the presence of behavioural impairment in patients with amyotrophic lateral sclerosis. Eur J Neurol.

[95] Aiello EN (2022). Cognition and motor phenotypes in ALS: a retrospective study. Neurol Sci.

[96] Olney NT, Spina S, Miller BL (2017). Frontotemporal dementia. Neurol Clin.

[97] Devenney EM, Ahmed RM, Hodges JR (2019). Frontotemporal dementia. Handb Clin Neurol.

[98] Qiu H et al (2021) ALS-associated mutation FUS-R521C causes DNA damage and RNA splicing defects*.* J Clin Invest 131(7) 10.1172/JCI149564 PMC8011883 33792567

[99] Fujii R (2005). The RNA binding protein TLS is translocated to dendritic spines by mGluR5 activation and regulates spine morphology. Curr Biol.

[100] Sahadevan S (2021). Synaptic FUS accumulation triggers early misregulation of synaptic RNAs in a mouse model of ALS. Nat Commun.

[101] Eyo UB (2021). Microglia provide structural resolution to injured dendrites after severe seizures. Cell Rep.

[102] Risher WC (2014). Astrocytes refine cortical connectivity at dendritic spines. Elife.

[103] Zhu YB (2016). Astrocyte-derived phosphatidic acid promotes dendritic branching. Sci Rep.

[104] Kia A (2018). Astrocytes expressing ALS-linked mutant FUS induce motor neuron death through release of tumor necrosis factor-alpha. Glia.

[105] Kerk SY (2022). Homozygous ALS-linked FUS P525L mutations cell- autonomously perturb transcriptome profile and chemoreceptor signaling in human iPSC microglia. Stem Cell Rep.

[106] Obrador E (2020). Oxidative stress, neuroinflammation and mitochondria in the pathophysiology of amyotrophic lateral sclerosis. Antioxidants (Basel).

[107] Meissner A (2015). Tumor necrosis factor-alpha underlies loss of cortical dendritic spine density in a mouse model of congestive heart failure. J Am Heart Assoc.

[108] Zou C (2016). Neuroinflammation impairs adaptive structural plasticity of dendritic spines in a preclinical model of Alzheimer’s disease. Acta Neuropathol.

[109] Liu Y (2017). TNF-alpha differentially regulates synaptic plasticity in the hippocampus and spinal cord by microglia-dependent mechanisms after peripheral nerve injury. J Neurosci.

[110] Doll DN (2015). Rapid mitochondrial dysfunction mediates TNF-alpha-induced neurotoxicity. J Neurochem.

[111] van Horssen J, van Schaik P, Witte M (2019). Inflammation and mitochondrial dysfunction: A vicious circle in neurodegenerative disorders?. Neurosci Lett.

[112] Gao J (2019). TDP-43 proteinopathy and mitochondrial abnormalities in neurodegeneration. Mol Cell Neurosci.

[113] Lopez-Domenech G (2016). Loss of dendritic complexity precedes neurodegeneration in a mouse model with disrupted mitochondrial distribution in mature dendrites. Cell Rep.

[114] Tsai YL (2020). ALS/FTD-associated protein FUS induces mitochondrial dysfunction by preferentially sequestering respiratory chain complex mRNAs. Genes Dev.

[115] Nakaya T, Maragkakis M (2018). Amyotrophic lateral sclerosis associated FUS mutation shortens mitochondria and induces neurotoxicity. Sci Rep.

[116] Salam S (2021). Identification of a novel interaction of FUS and syntaphilin may explain synaptic and mitochondrial abnormalities caused by ALS mutations. Sci Rep.

[117] Tradewell ML (2012). Arginine methylation by PRMT1 regulates nuclear-cytoplasmic localization and toxicity of FUS/TLS harbouring ALS-linked mutations. Hum Mol Genet.

[118] Deng J (2015). FUS interacts with HSP60 to promote mitochondrial damage. PLoS Genet.

[119] Uranishi H (2001). Involvement of the pro-oncoprotein TLS (translocated in liposarcoma) in nuclear factor-kappa B p65-mediated transcription as a coactivator. J Biol Chem.

[120] Shelkovnikova TA (2019). Antiviral immune response as a trigger of FUS proteinopathy in amyotrophic lateral sclerosis. Cell Rep.

[121] Ajmone-Cat MA (2019). Increased FUS levels in astrocytes leads to astrocyte and microglia activation and neuronal death. Sci Rep.

[122] Krysko DV (2011). Emerging role of damage-associated molecular patterns derived from mitochondria in inflammation. Trends Immunol.

[123] Lin MM (2022). Mitochondrial-derived damage-associated molecular patterns amplify neuroinflammation in neurodegenerative diseases. Acta Pharmacol Sin.

