# Supplementary material for: Neuronal dysfunction caused by FUSR521G promotes ALS-associated phenotypes that are attenuated by NF-κB inhibition
Source: Acta Neuropathol Commun. 2023 Nov 16;11:182. doi: 10.1186/s40478-023-01671-1 (PMC10652582; doi:10.1186/s40478-023-01671-1)
Supplement: Supplementary file 1 — Additional file 1. Supplementary methods and figures: Methods. Material and methods for supplemental data. Figure legends. Figure legends for supplemental figures. Fig. S1. hFUS co-staining with markers for astrocytes or microglia. Fig. S2. Excision and expression analysis of hFUS in the brain and spinal cord of hFUSR521/Syn1 mice. Fig. S3. hFUSR521/Syn1 mice present persistent cognitive impairment, along with progressive, age-dependent motor dysfunction. Fig. S4. No sex-dependent differences are observed in hFUShFUSR521/Syn1 mice. Fig. S5. Progressive loss of motor neurons and NMJ denervation in aged hFUSR521/Syn1 mice. Fig. S6. No defects in spinal motor neurons and NMJ in 6-month-old hFUSR521/Syn1 mice. Fig. S7. IMS-088 treatment attenuates glial activation in the cortex of hFUSR521/Syn1 mice. [file 40478_2023_1671_MOESM1_ESM.docx]

**SUPPLEMENTARY INFORMATION**

**Methods**

**Behavioral testing**

Behavioral tests were performed as previously described [42] and as detailed below. Longitudinal testing of mice was performed in order to capture the progressive cognitive and motor decline that is observed in ALS/FTD. In this study, the same mice were used for all time points. The number of mice tested for 2, 4, 8 and 12 months of age included 14 (6M:8F) littermate controls (+/+;+/+, +/+;+/Cre, Tg/+;+/+) and 12 (5M:7F) hFUS^R521G/Syn1^ transgenic (Tg/+;+/Cre) mice. The number of mice tested at 18 months of age included 10 (5M:5F) littermate controls (+/+;+/+, +/+;+/Cre, Tg/+;+/+) and 9 (4M:5F) hFUS^R521G/Syn1^ transgenic (Tg/+;+/Cre) mice.

Catwalk test. The paws of each test mouse were painted in two different colours using non-toxic paint. Animals were placed at one end of a catwalk structure lined with a 7.5 x 25 cm piece of paper, with the home cage of the test mouse placed at the other end of the catwalk. Paw marks are made by each mouse on the paper as they walk in the direction of their home cage. The distance between the tracks were measured and indicated as hindlimb stride length and base of support, and forelimb stride length and base of support.

**FUSR521G expression analysis**

Brain and lumbar spinal cord sections from hFUS^R521G/Syn1^ and littermate control mice were stained with anti-human-FUS (hFUS), anti-NeuN, anti-GFAP and anti-CD11b. Tissue imaging was performed using a Zeiss LSM710 inverted confocal and imaged as z-stacks (4μm steps per stack) tiled with a 10% image overlap. To determine hFUS level of expression the signal intensity quantification of hFUS was performed on maximum intensity projection images and analysed in Fiji ImageJ using the signal intensity measuring tool. hFUS signal intensity for each image was normalized to area. Spinal cord analysis of hFUS include ventral and dorsal neurons and cortex analysis of hFUS include the motor cortex, medial frontal cortex and lateral cortex (including the somatosensory cortex). No background subtraction was applied in the analysis. A total of 3 tissue sections were quantified per animal from 3 biological replicates per group.

Mouse and human FUS proteins were resolved by western blotting from cortex and whole spinal cord homogenates as described in the material and methods section in the main text. Mouse and human FUS signal intensities were quantified using Image Studio Lite software (version 5.2) and normalized to the signal intensities of β-Actin from 3 biological replicates and Ponceau from 2 biological replicates.

**FUSR521G excision analysis**

Brain and lumbar spinal cord sections from hFUS^R521G/Syn1^ mice were stained with anti-hFUS and anti-NeuN to label neurons positive for the transgene. Confocal images were captured as described above using a Zeiss LSM710 inverted confocal and imaged as z-stacks (1-2μm steps per stack). To determine the transgene percentage of excision, NeuN positive cells and NeuN positive cells co-stained with hFUS signal were counted using the counting tool from Fiji ImageJ. Results were expressed as a percentage of NeuN cells co-stained with hFUS. Tissue sections were quantified per animal from 3 biological replicates per group.

**Nissl staining**

Sectioned tissues were mounted onto slides and incubated in a 1:1 alcohol and chloroform solution overnight. The next day, tissue sections were dehydrated through incubation with 100% and 95% alcohol solution for 1 min each. Sections were stained with 0.1% cresyl-violet solution for 1-5 min at RT, rinsed with distilled water for 2 min and distained with 95% and 100% alcohol solution for 5 min each. Finally, sections were incubated in Xylene for 5 min and mounted with Permount (Fisher Scientific, SP15-100). Brightfield images were taken with a Leica DM5000 B and a 10X objective and motor neurons with a cell body diameter >100μm were quantified from 7-10 independent tissue sections from the ventral horn of the lumbar spinal cord from 3-4 biological replicates per group.

**Statistics**

All statistical analyses were performed using GraphPad Prism 8 (GraphPad, San Diego, CA). One-way and two-way ANOVA analyses used the Bonferroni post-hoc test. All values given in the text and figures indicate mean ± standard error of the mean (SEM). The level of significance was specified as follows: * p<0.05, ** p<0.01, ***p<0.005 and **** p<0.001.

**Figure legends:**

**Figure S1.** hFUS co-staining with markers for astrocytes or microglia. (**a**) Brain and (**b**) spinal cord tissues from CTL and hFUS^R521G/Syn1^ mice co-stained with anti-hFUS, anti-GFAP (astrocyte marker) and anti-CD11b (microglia marker) show no expression of hFUS in cells positive for CD11b or GFAP.

**Figure S2.** Excision and expression analysis of hFUS in the brain and spinal cord of hFUS^R521/Syn1^ mice. (**a**) Brain and (**b**) spinal cord tissues from CTL and hFUS^R521G/Syn1^ mice co-stained with anti-hFUS and anti-NeuN. Tiled images show the transgene is equally distributed throughout the tissues. Inserts show co-staining of hFUS with NeuN. (**c**) The percentage of NeuN positive neurons co-stained with hFUS in the cortex and spinal cord of hFUS^R521G/Syn1^ mice show nearly 100% of neurons express hFUS. (**d**) Quantification of hFUS signal intensity in mice cortex and spinal cord shows no significant differences in hFUS expression between these tissues. (**e**) Quantification of hFUS signal intensity in different brain regions shows no differences in the transgene expression. (**f**) Quantification of hFUS signal intensity in the ventral and dorsal horns of the spinal cord shows no differences in the transgene expression. (**g**) Immunoblot of lysates from CTL and hFUS^R521G/Syn1^ mice. Proteins are detected with antibodies against total FUS (tFUS) and the loading control (**h**) β-Actin or (**i**) Ponceau. The lower molecular weight band in the immunoblot corresponds with endogenous mouse FUS (mFus) and the higher molecular weight band in the immunoblot corresponds with human FUSR521G protein (hFUS). Quantitative data are mean ± SEM. Statistics uses an unpaired Student’s *t*-test for comparison between two groups. not significant (ns).

**Figure S3.** hFUS^R521/Syn1^ mice present persistent cognitive impairment, along with progressive, age-dependent motor dysfunction. (**a**) Novel object recognition test performed at the ages of 2, 4, 8 and 18 months. Novel object (N) and familiar object (F). (**b**) Passive avoidance test carried out by hFUS^R521G/Syn1^ and control mice at the ages of 2, 4, 8 and 18 months. (**c**) Wire hanging test performed at 2, 4, 8 and 18 months. (**d**) Rotarod test performed at 2, 4, 8 and 18 months. (**e**) Catwalk gait test performed at 8, 12 and 18 months. (2-12 months of age: n=12 hFUS^R521G/Syn1^ and n=14 CTL mice/group; 18 months of age: n=9 hFUS^R521G/Syn1^ and n=10 CTL mice/group). Quantitative data are mean ± SEM. Statistics uses an unpaired Student’s *t*-test for comparison between two groups. **p* < 0.05, ***p* < 0.01, ****p* < 0.005, *****p* < 0.001 and not significant (ns).

**Figure S4.** No sex-dependent differences are observed in hFUS^R521/Syn1^ mice. Cognitive and motor tests were performed on 8-months-old hFUS^R521/Syn1^ mice and their littermate controls (CTL). (**a**) Novel object recognition test shows male and female hFUS^R521/Syn1^ mice do not recognize the novel object unlike age and sex matched controls. Novel object (N) and familiar object (F). (**b**) Passive avoidance test shows male and female hFUS^R521/Syn1^ mice do not have a latency time to entering the dark chamber when compared to age and sex matched controls. (**d**) Rotarod test shows male and female hFUS^R521/Syn1^ mice spend less time on the rotarod on day 2 of behavior testing when compared to age and sex matched controls. (Males: n=5 hFUS^R521G/Syn1^ and n=6 CTL; Females: n=7 hFUS^R521G/Syn1^ and n=8 CTL mice/group). Quantitative data are mean ± SEM. Statistics uses an unpaired Student’s *t*-test for comparison between two groups. **p* < 0.05, ***p* < 0.01, ****p* < 0.005 and not significant (ns).

**Figure S5.** Progressive loss of motor neurons and NMJ denervation in aged hFUS^R521G/Syn1^ mice. (**a**) Nissl staining on spinal cords of littermate control (CTL) and hFUS^R521G/Syn1^ mice at the indicated ages. (**b**) Quantification of the number of motor neurons in the ventral horn of the spinal cord of mice. (**c**) The spinal cord of CTL and hFUS^R521G/Syn1^ mice stained with anti-ChAT (motor neuron marker) and anti-NeuN. (**d**) Quantification of spinal motor neuron co-stained with ChAT and NeuN. (**e**) Neuromuscular junctions from gastrocnemius stained with α-bungarotoxin-647 (BTX) and anti-synaptophysin (SYP). (**f**) Quantification of neuromuscular junctions co-stained with BTX and SYP. Quantitative data are mean ± SEM. Statistics uses an unpaired Student’s *t*-test. **P<0.01, ***P<0.001, ****P<0.0001, not significant (ns).

**Figure S6.** No defects in spinal motor neurons and NMJ in 6-months-old hFUS^R521G/Syn1^ mice. (**a**) Nissl staining on spinal cords of control (CTL) and hFUS^R521G/Syn1^ mice at 6 months of age. (**b**) Quantification of the number of motor neurons in the ventral horn of the spinal cord of mice. (**c**) Neuromuscular junctions from gastrocnemius stained with α-bungarotoxin-647 (BTX) and anti-synaptic vesicle glycoprotein 2A (SV2) show normal co-staining of BTX and SV2 at neuromuscular junctions in 6-months-old hFUS^R521G/Syn1^ mice. Quantitative data are mean ± SEM. Statistics uses an unpaired Student’s *t*-test. not significant (ns).

**Figure S7.** IMS-088 treatment attenuates glial activation in the cortex of hFUS^R521/Syn1^ mice. (**a**) Cortex staining with anti-GFAP and anti-Iba1 in 8-months-old mice treated IMS-088 (IMS) or vehicle (veh). (**b**) Signal intensity (S.I) for GFAP and Iba1 show IMS-088 inhibits activation of astrocytes and microglial in treated FUS transgenic mice compared to vehicle treated mice. Values from each group are expressed as mean ± SEM. Statistics uses a one-way ANOVA for multiple group comparisons. *****p* < 0.001.

**Supplementary figures**


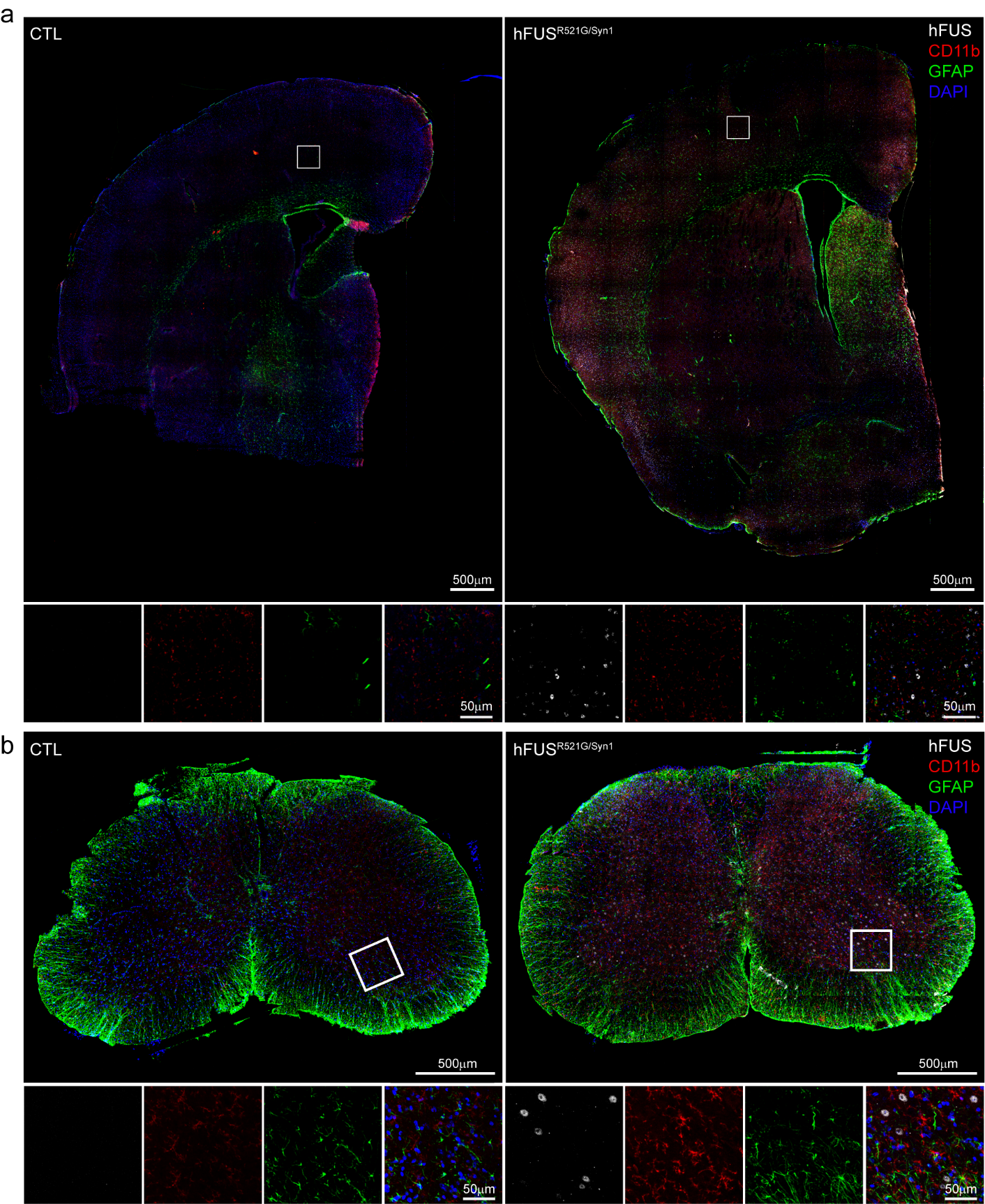


**Figure S1.**


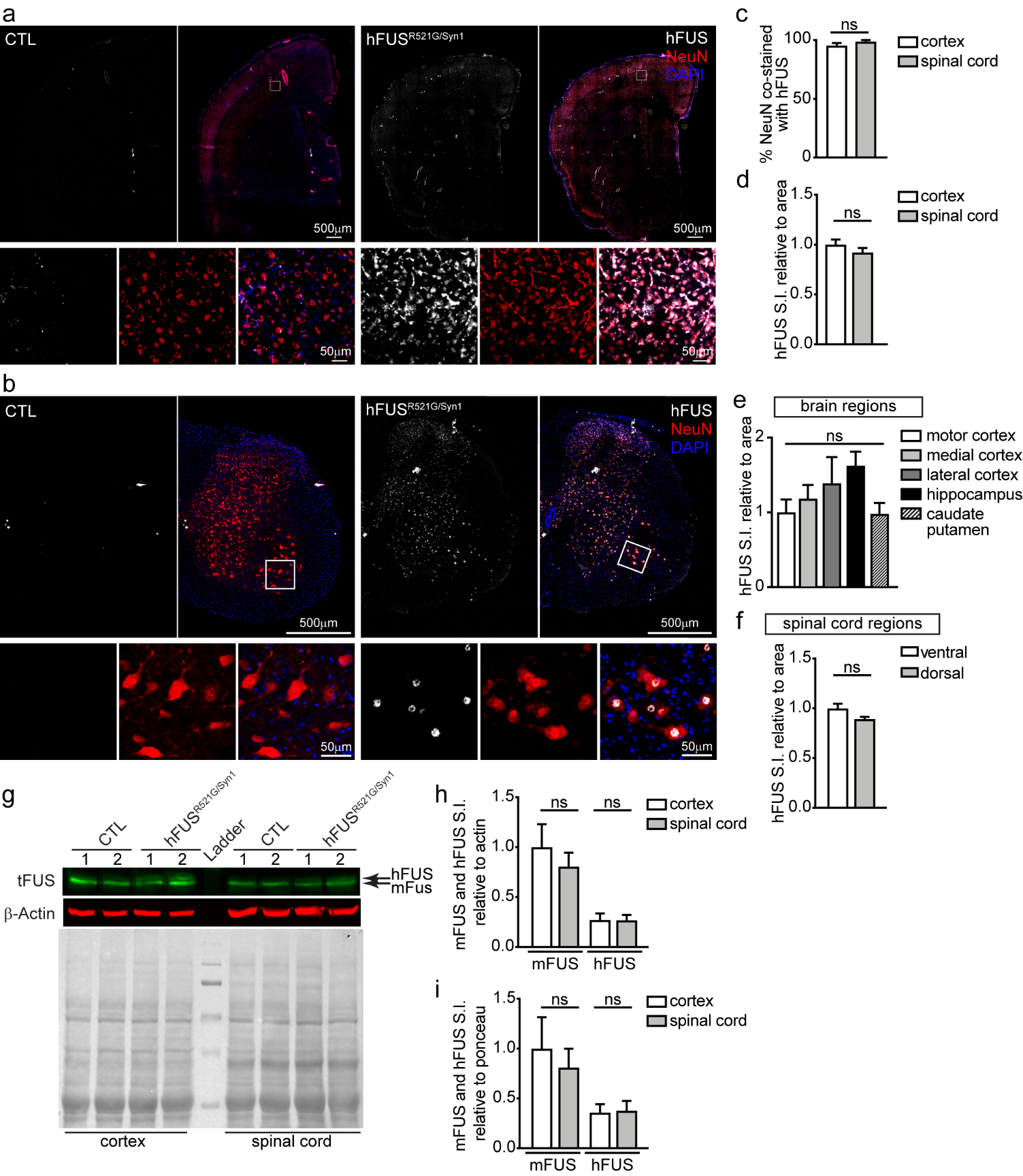
**Figure S2.**


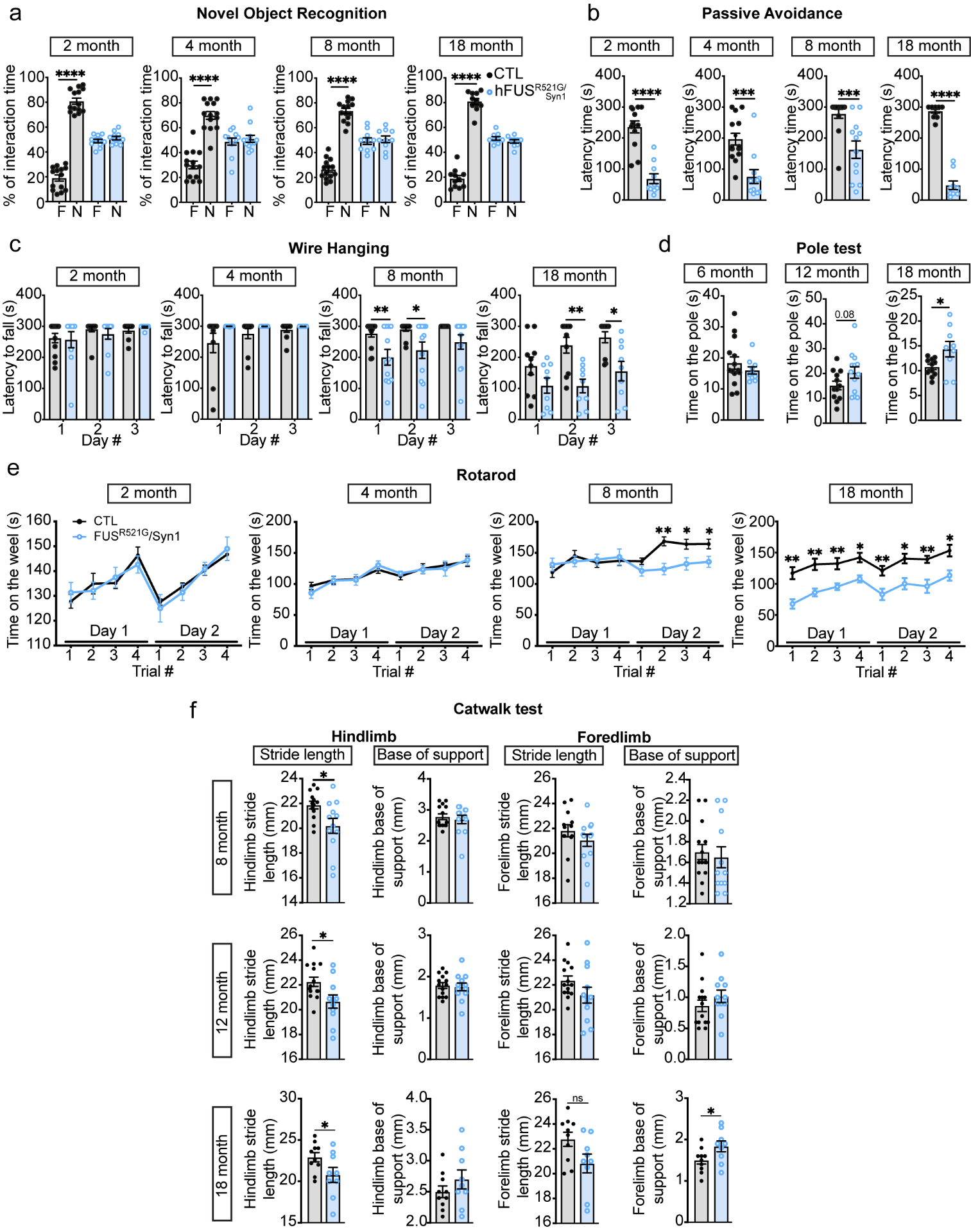
**Figure S3.**


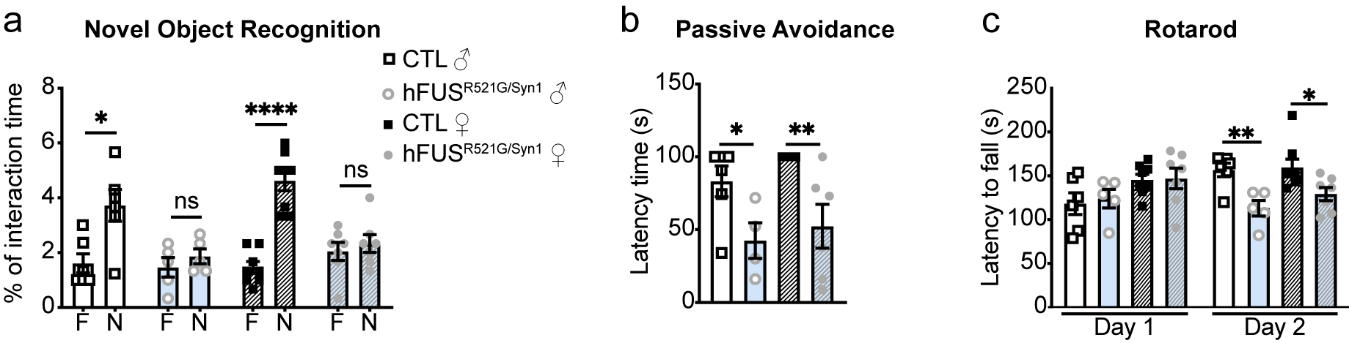


**Figure S4.**


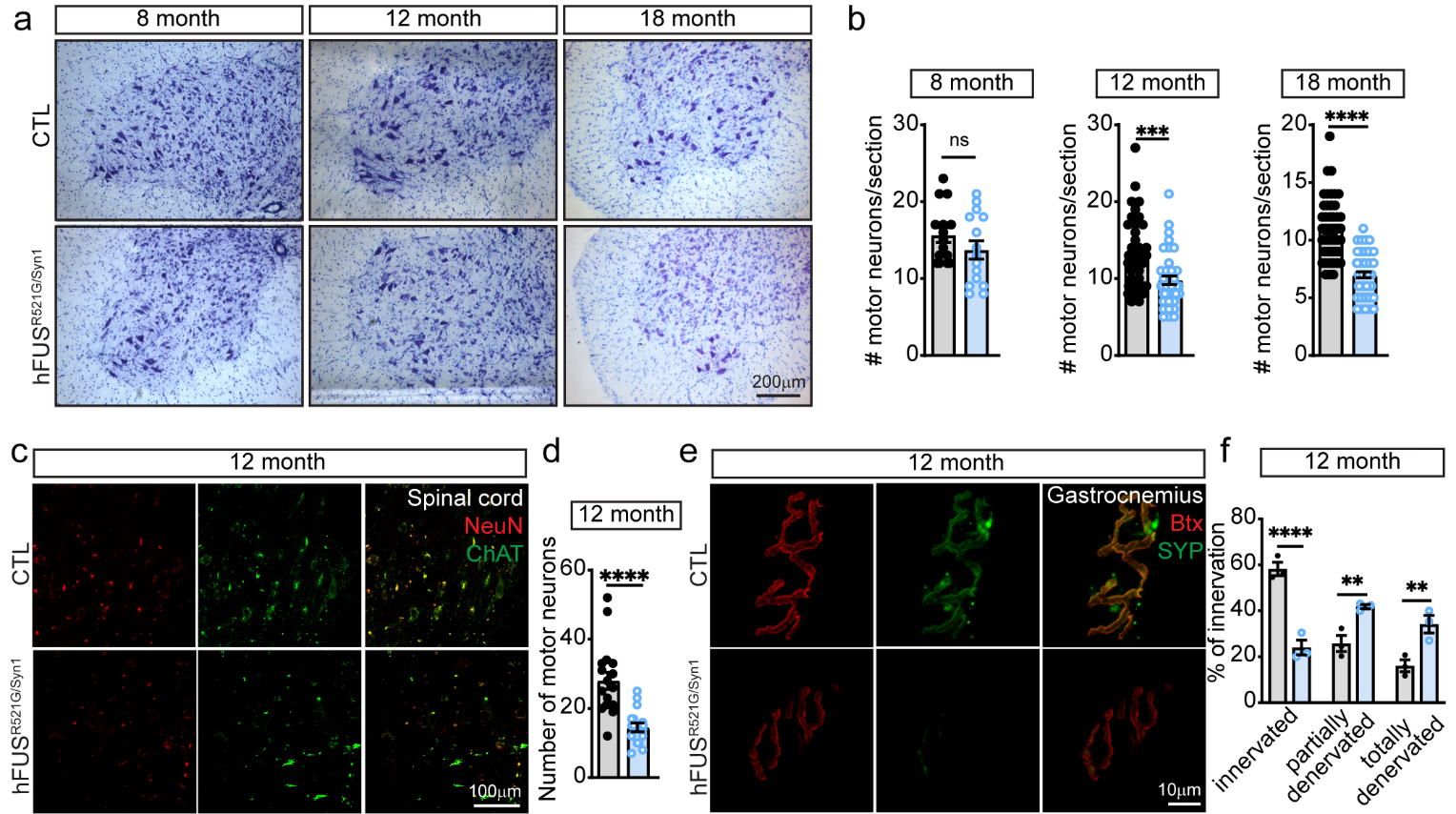


**Figure S5.**


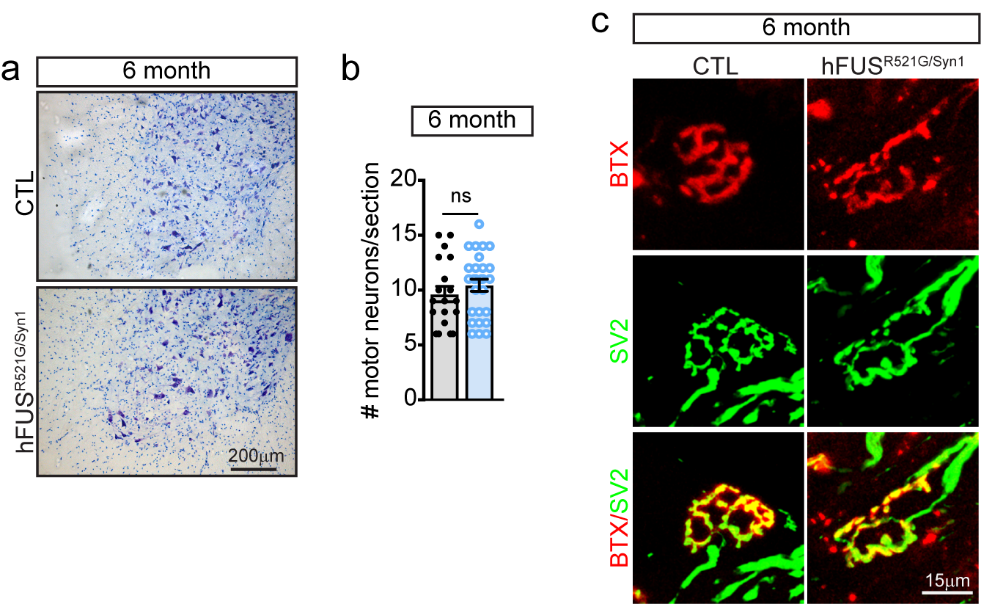


**Figure S6.**


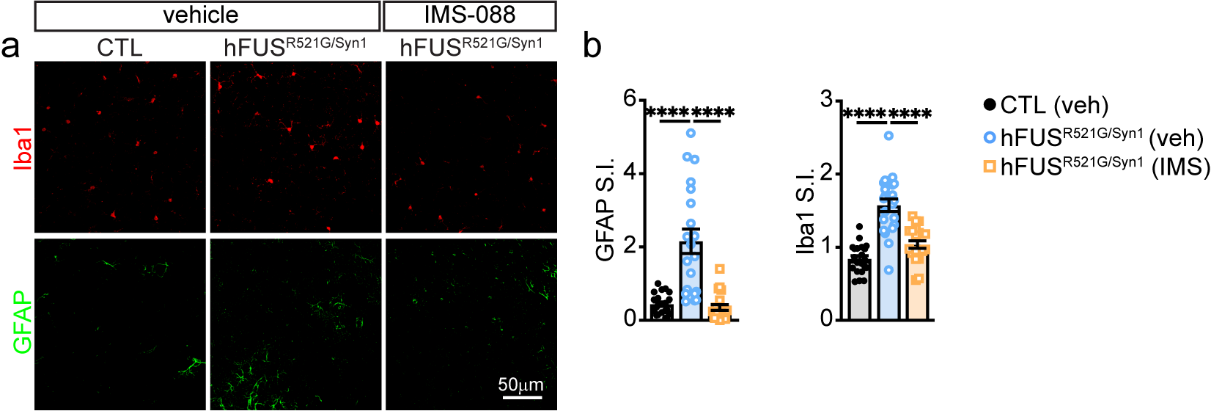


**Figure S7.**
